# Evolving the DarTwin evolution language

**DOI:** 10.1007/s11334-026-00647-3

**Published:** 2026-09-23

**Authors:** Øystein Haugen, Martin Arthur Andersen, Stefan Klikovits, Francis Bordeleau, Joost Mertens, Joachim Denil

**Affiliations:** 1 Department of Computer Science and Communication, Østfold University of Applied Sciences, B R A veien 4, 1757 Halden, Norway; 2 Institute for Business Informatics - Software Engineering, Johannes Kepler University, Altenberger Strasse 69, 4040 Linz, Austria; 3 Department of Software Engineering and Information Technology, École de Technologie Supérieure (ÉTS), Montreal, Canada; 4 Faculty of Applied Engineering: Electronics and ICT, University of Antwerp, Groenenborgerlaan 171, 2020 Antwerp, Belgium; 5 Flanders Make @UAntwerpen, University of Antwerp, Groenenborgerlaan 171, 2020 Antwerpen, Antwerp Belgium

**Keywords:** Evolution, Domain-specific language, Digital twin, SysML v2, MetaEdit+, DarTwin

## Abstract

DarTwin is a notation for describing and planning evolution of digital twin systems. Originally conceived as a graphical notation, DarTwin has evolved into a domain-specific language formally defined in SysML v2. This evolution exposed challenges related to graphical notation support, tooling interoperability, and co-evolution between notation, language definition, and modelling environments. We experienced that SysML v2 tooling was not adequate to provide our DarTwin graphical notation even with some post-processing. The SysML v2 definition of DarTwin did, however, motivate improvements of the DarTwin DSL. To provide fruitful DarTwin graphical notation and still maintain the formal definition in SysML v2 we demonstrate how domain-specific tooling (MetaEdit+) helps improving the graphic editing and rendering support and thus achieve a more effective and intuitive use of DarTwin. By generating SysML v2 from MetaEdit+ we preserve the connection between the adequate graphical notation and the precision and formality of DarTwin. The combined DarTwin tooling made the methodology to apply general evolution transition patterns (DarTrans) more formal and precise while still intuitive through graphic notation using colour and dashes. This in turn motivated enhancing the DarTwin language by adding concepts related to the evolution transitions to facilitate discovering and documenting the transitions.

## Introduction

This article is about evolution on two interrelated matters: We defined a graphical notation for describing the evolution of Digital Twin (DT) systems called DarTwin  [36], named as a combination of Darwin (the scientist famous for his evolutionary theory) and the target of the language by the term “Twin”. This article describes how the DarTwin language itself evolved to achieve precision while keeping the original intuitive graphical notation. To achieve precision we defined DarTwin as a DSL in SysML v2 [24] and hoped this would also make it possible to apply SysML v2 tools for graphic rendering. Realizing that we could not achieve adequate DarTwin rendering with current SysML v2 tooling, we explore a multi-tool environment including both SysML v2 and a dedicated DSL tool (MetaEdit+). Finally, we show how this multi-tool environment opens up for further improvements of the DarTwin DSL.

SysML v2  [42] [39], a successor to the widely used SysML v1 language, heralds a new era of systems engineering, promising a streamlined systems modelling experience, more integrated features, and improved extension capabilities. Severing its dependency on SysML v1 ’s underlying UML language, SysML v2 builds on the Kernel Modeling Language (KerML) [40], which enables semantically and syntactically rich and well-defined mechanisms for integrating domain-specific concepts.

***DarTwin as SysMLv2-based DSL***

This article is an extension of our previous work [24], where we describe the implementation of a DSL for DarTwin. DarTwin DSL applies the DSL capabilities of SysML v2. Our previous work explored three assumptions regarding the use of SysML v2 as a foundation for DarTwin DSL.

- By specifying DarTwin DSL using SysML v2 DSL mechanisms, we obtain a precise DarTwin language with clearly-defined concepts and semantics.
- By building on SysML v2, DarTwin DSL should directly integrate with other SysML v2 models.
- By developing DarTwin DSL using SysML v2, the DSL would automatically benefit from the latest generation of SysML v2 tooling, providing convenient modelling support.

Our findings showed that

- The implementation of DarTwin in SysML v2 not only made the language precise, but it also resulted in the discovery of some necessary improvements to DarTwin.
- The DarTwin DSL integrated directly with SysML v2 and our DarTwin models in textual form were checked through the SysML v2 Pilot Implementation  [41] (an open-source implementation of SysML v2 that is maintained continuously by the standardization community).
- We also observed that existing SysML v2 tools could not properly support the visual aspects of DarTwin DSL, pointing towards several shortcomings. Given that the DarTwin originated as a graphic notation [36] to support the overview and communication on DT systems, where DTs play a significant role, we found that the SysML v2 tools did not give a graphic rendering that was as intuitive as our original DarTwin notation. Currently, SysML v2 does not provide means to specify domain-specific rendering styles, but this may come in the future. The SysML v2 tools also had significant general shortcomings.

This revealed a tension between maintaining the formal precision of SysML v2 and preserving the intuitive graphical qualities that originally motivated DarTwin.

***Contributions***

The central challenge addressed in this article is therefore how to preserve the formal precision and interoperability benefits of SysML v2 while recovering the intuitive graphical notation that originally motivated DarTwin.

Our further aims are to explore:

1. How dedicated DSL tooling can support the intended DarTwin graphical notation while preserving compatibility with SysML v2.
2. Whether graphical tooling simplifies applying general DarTrans transition patterns.
3. How enhanced graphical tooling may motivate further evolution of the DarTwin language itself.

In this article, we expand on the discovered shortcomings and explore the use of MetaEdit+  [28], a dedicated DSL-workbench, to improve the language support of DarTwin. We selected MetaEdit+  [28] based on its mature DSL capabilities and our prior experience with the platform.

We combine two technologies by developing support for graphical DarTwin DSL in MetaEdit+, and a generator in MetaEdit+ to create textual SysML v2 and thus maintaining the close relation between DarTwin and SysML v2, thereby allowing for integration in typical systems engineering workflows.

Our findings show that

1. The MetaEdit+ workbench is capable of creating diagrams very similar to our manually crafted DarTwin diagrams (e.g. using TikZ [47]), while meeting requirements of the underlying DarTwin DT systems evolution notation [36]. Indeed, by using MetaEdit+, the DSL becomes more intuitively useful than using SysML v2 ’s textual syntax. In particular, we were able to use features that are unavailable in the current SysML v2 tooling, such as using colour to depict changes in DarTrans. The precision of SysML v2 was kept through generating SysML v2 and checking it on the SysML v2 Pilot Implementation.
2. We performed an example where the application of the pattern methodology with textual SysML v2 was one approach and applying graphic MetaEdit+ was the other approach. Our own assessment was that the graphic approach was simpler and more intuitive.
3. When doing examples with our transition methodology using DarTrans diagrams, we realized the need for documenting triggers for initiating an evolution and also a way to document the intended strategy. The descriptions of triggers and strategies on generic pattern DarTrans may also serve to help the selection of evolution pattern. The evolution of DarTwin DSL fits well with MetaEdit+ ’s language capabilities, and we found that MetaEdit+ facilitates the creation of graphical mock-ups of suggested changes to the language.

This article is structured as follows. Section 2 provides the necessary background, covering the DarTwin notation, its prior formalization as an embedded DSL in SysML v2, and the graphical shortcomings identified in current SysML v2 tooling. Section 3 describes the implementation of DarTwin in MetaEdit+, including the GOPPRR-based language definition and the experiences encountered during that process. Section 4 compares the graphical output produced by MetaEdit+ against that of three SysML v2 tools, using the Strawberry Cultivation System as a reference example. Section 5 demonstrates the application of DarTrans evolution patterns in both SysML v2 and MetaEdit+, employing a gantry crane case study to evaluate the relative merits of textual and graphical approaches. Section 6 outlines prospective enhancements to DarTwin  3.0, including the incorporation of trigger and strategy concepts into DarTrans diagrams. Section 7 frames the co-evolution challenges observed across the DarTwin toolchain using established taxonomies from the literature. Section 8 situates the contribution within related work on system evolution, model-driven engineering, and DSL co-evolution. Finally, Section 9 concludes the article and identifies directions for future research.

## Background

In this section, we first describe the foundation of our research, namely the DarTwin notation. Second, we provide details of our previous development of DarTwin DSL and its implementation in SysML v2. Finally, we outline the shortcomings of the current generation of SysML v2 tools in terms of graphical DSL support using DarTwin DSL as an example.

### DarTwin notation

By combining continuous information exchange between a digital representation of an actual system, DTs  [21] enable the provision of powerful services such as predictive maintenance, advanced monitoring, and model- and data-driven optimizations. Thereby, live data is continuously streamed from the Actual Twin (AT) to the DT, and used to improve various aspects of the AT.

We refer to the combination of an AT with one or several DTs as a DT system. Thereby, each DT in a DT system may serve a specific (ideally non-conflicting) purpose.

Like any other engineered system or software artefact, a DT system is inherently subject to continuous evolution [36] that may manifest across all of its constituent aspects [48]. Drawing on prior work [36], three distinct categories of DT system evolution can be identified:

1) Changes in the AT or its operational environment, 2) Evolutions of the DTs themselves, and 3) Shifts in a DT ’s *purpose* and/or intended goals.

Type 1) resembles classical systems engineering challenges, encompassing phenomena such as physical degradation (e.g. due to wear and tear), the modification or replacement of system components, or changes in the AT ’s surrounding environment. Type 2) pertains to improvements, refinements, or corrections applied to the DT, typically arising from sustained operational evaluation over time. Type 3) commonly emerges from evaluating the system in deployment and identifying opportunities that were not apparent during the initial design phase. It may equally arise from newly introduced requirements or from shifting priorities amongst business stakeholders. In practice, evolutionary changes frequently comprise a combination of the aforementioned types.

To mitigate the risk of invalidating the DT system or causing damage to the AT in the face of system evolution—and equally to manage the inherent complexity of the evolutionary process itself—such evolution ought to be approached in a planned, documented, and systematic manner.

DarTwin  [36] is a graphical notation to express the evolution of DT systems. It was developed as a description and communication means to address the continuous evolution of DT systems. In [36] we argue that this aspect is of particular importance and illustrate this using two use cases: a smart home running example and a gantry crane validation system.

**Fig. 1:**
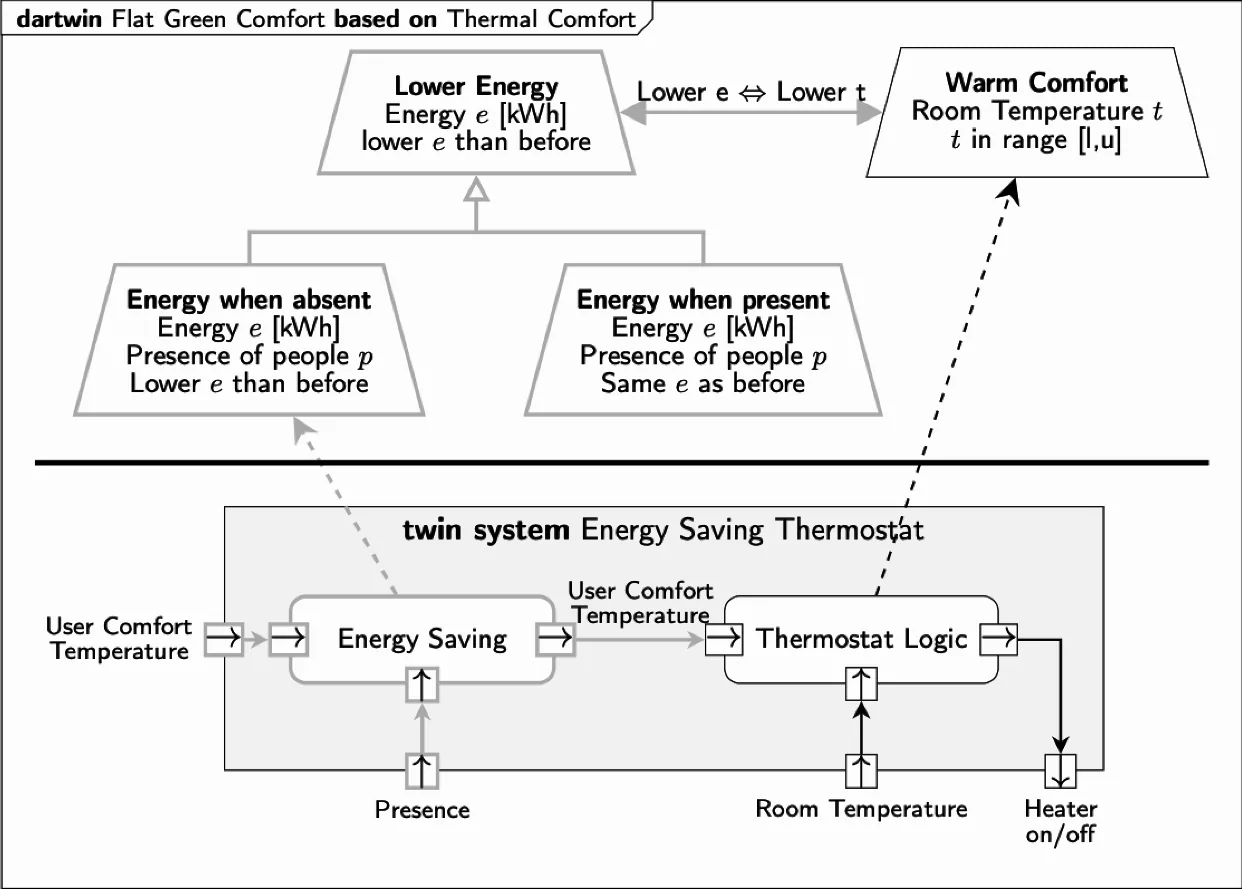
DarTwin notation example (from [36])

DarTwin was deliberately conceived to provide an abstract, graphical overview of DTs and their associated goals. Within the DarTwin graphical view (Fig. 1), goals are rendered in the upper region, whilst the composite structure of the DTs and their interconnections occupies the lower region, with a horizontal bar demarcating the two.

The illustrative instance in Fig. 1 depicts the modelling of DT *goals* and their interrelations (top half), as well as the manner in which these goals are realized by an underlying architecture of DTs within a DT system, interconnected via *ports* (below the separation line).

### DarTwin in SysML v2

When a notation seems useful, the next step is often to develop a DSL and provide adequate tooling for description and analysis. A DSL differs from a notation by giving more precision and formality. From the process of making the notation a language, issues of confusion and ambiguity will typically emerge. This is the first phase of creating a DSL and we built upon the DarTwin notation and developed DarTwin DSL. We implemented DarTwin as an *embedded DSL* in SysML v2, and this resulted in improvements [24].

By defining DarTwin using SysML v2, it makes the language precise, offering a clear syntax and semantics that are defined through SysML v2 ’s language extension mechanisms such that DarTwin can be seen as an extension of SysML v2. That means that tools for SysML v2 are applicable for DarTwin. By integrating DarTwin with the (likely) de-facto standard in systems engineering, our approach increases the applicability of the DarTwin DSL and facilitates its adoption. This also allows reusing SysML v2 ’s language infrastructure, library system, model encoding, etc.

#### Defining embedded DSLs in SysML v2

The release of SysML v2  [42] provides developers with a capable tool to design and analyse their systems. Notably, the language is built for extensibility and enables the creation of custom keywords and concepts. This enables users to create domain-specific models with special concepts that are more intuitive for the domain experts, but still defined precisely through SysML v2. This combination of domain concepts and SysML v2 is what we shall explore for the DarTwin notation.

Two mechanisms are essential for creating a DSL: specialization and redefinition. Specialization (denoted textually by ) allows a model element to inherit properties from another element while potentially adding new characteristics. Redefinition (denoted by ) enables a form of inheritance where the specialized element can modify or override the characteristics of its parent. These mechanisms from object orientation combined with a construct to map user defined keywords to inherited concepts create the foundation for language engineering in SysML v2, allowing domain-specific concepts to be formally anchored in the SysML v2 language’s core.

#### Defining DarTwin DSL in SysML v2

**Fig. 2:**
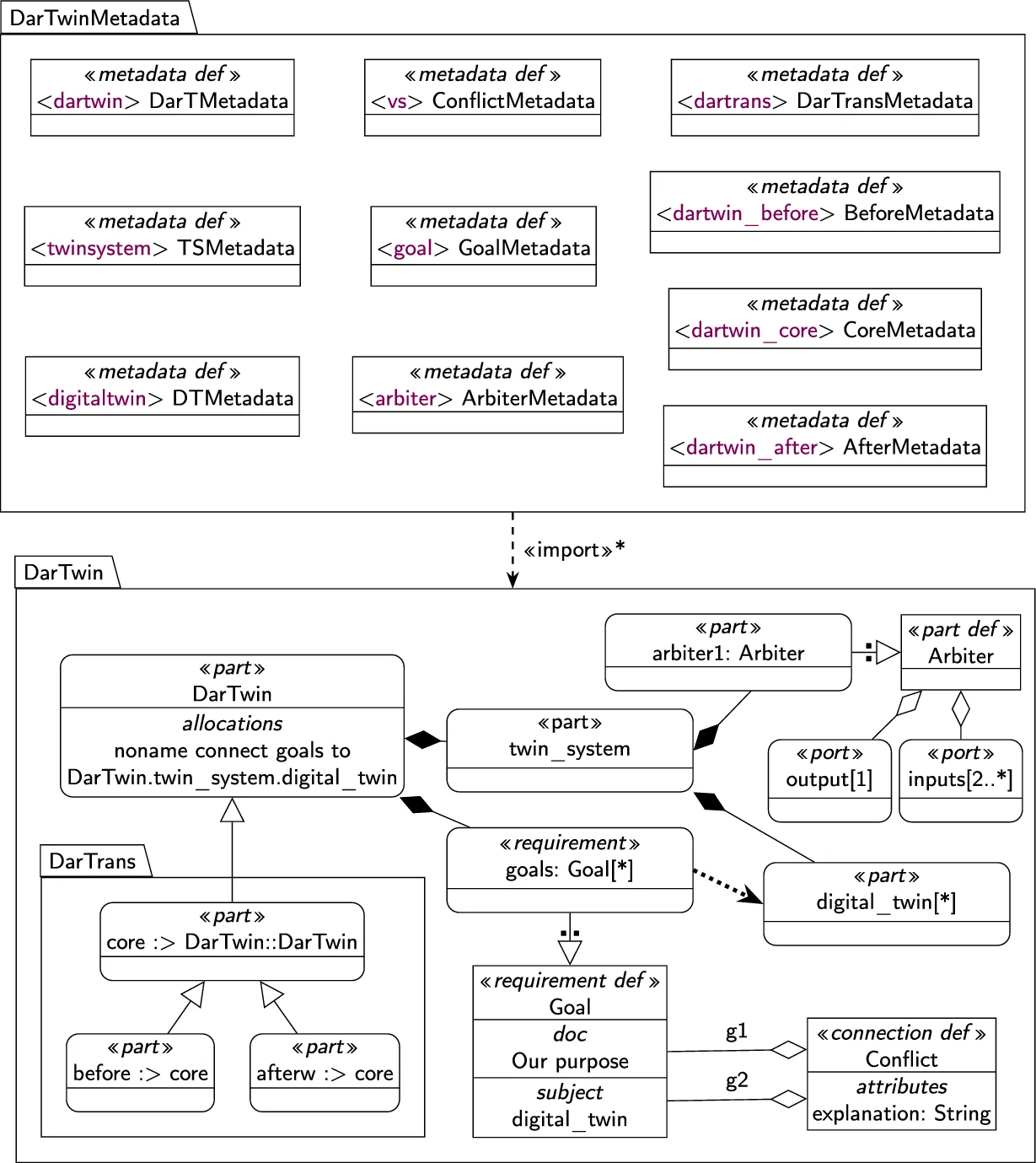
DarTwin metamodel expressed as SysML v2 libraries (adapted from [24] with a minor change)

The DarTwin DSL definition consists of a metamodel comprised by a library defined in SysML v2 of the DarTwin concepts, supplemented by the declaration of the corresponding DarTwin keywords in SysML v2. Figure 2 shows the conceptual metamodel of DarTwin concepts at the bottom, the matching meta-definitions for keyword declarations (e.g. , , ) at the top.

In the metamodel the concepts of DarTwin are depicted in the SysML v2 version of a class diagram. A  represents that a DT system contains (depicted by a black diamond) a  and a set of  elements. The  contains a set of  definitions. We use an allocation relation to connect each  with one or more  elements to represent what goals the DTs intend to satisfy. The AT can merely be represented by plain SysML v2 ports on the edge of the  and is therefore not shown in the metamodel^1^.

The  represents a DarTrans transition by three  instances, named , ,  The two latter ones inherit the core which represents those elements that do not change in the transition. We detail this in Sect. 2.2.3.

**Fig. 3:**
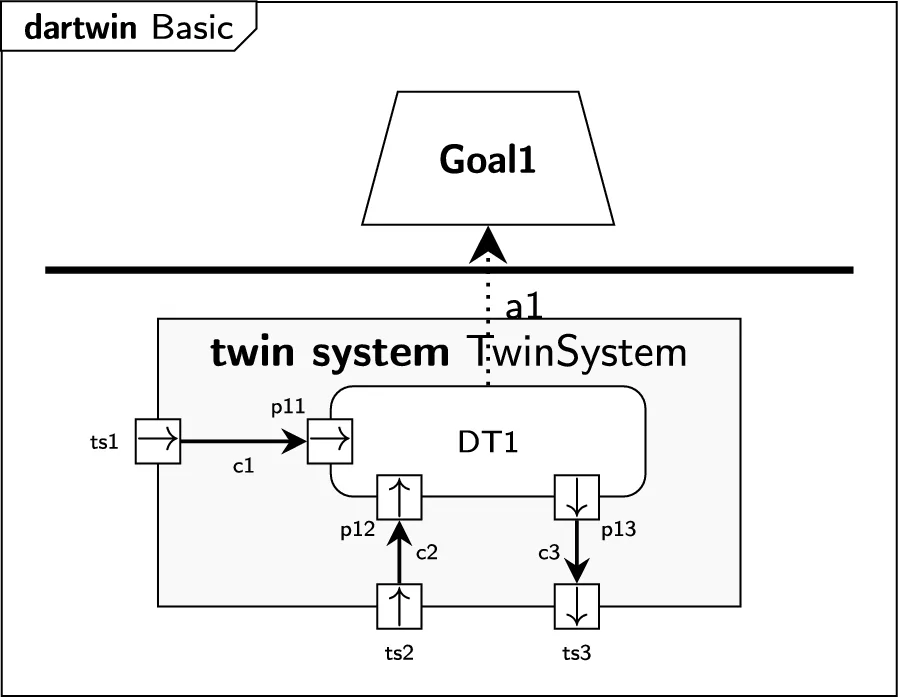
DarTwin Basic with explicit names on ports and connections.

Using these domain-specific definitions, we can model DarTwin in our textual DSL as shown in Listing 1, which is based on the *Basic* DT system that was originally shown in [36]. In Fig. 3 we explicitly provide the names of the ports and connections to make the correspondence to Listing 1 easier.

**Figure:**
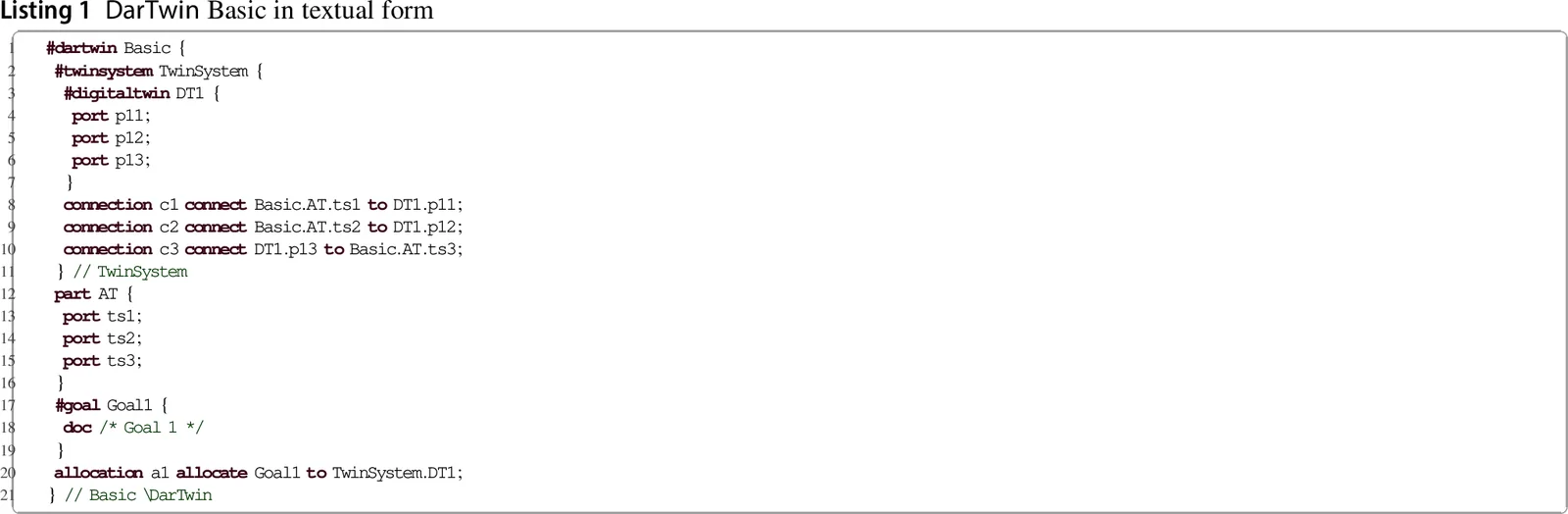

#### Defining DarTrans transitions

While the purpose of DarTwin is to describe the evolutions of DT systems, we note that the DarTwin in Listing 1 defines an initial DT system starting point. To define the evolution transition more precisely, we extended DarTwin DSL to include the concept of DarTrans transitions [24]. This extension to the original DarTwin notation defines the capacity to add and remove elements. Thereby, DarTrans enhances SysML v2, which does not natively support self-modifying operations. Technically, we employ SysML v2 ’s specialization for our purposes. In practice the transition must be implemented by editing the model.

A   transition in DarTwin DSL is a description of an evolution from an initial    to an evolved .

 transitions represent three interrelated models that, in combination, constitute the transition.

: all the common elements that do not change during the evolution.
specializes  and lists the elements that will be *deleted* in the evolution. Together with the inherited elements from  they represent the starting point of evolution.
specializes  and lists the *added* elements of the evolution.
  Note that *changing* or *updating* an element is modeled as a combination of deleting the old and updating the new version. Thus, a modified element should be present in both  and . Together with the inherited elements from  it represents the result of the evolution.

Visually, a  is depicted similarly to a  model, with the difference that changes in components and connections are drawn in orange colour. Specifically,  shows deleted elements using dashed lines, while elements that are updated or added by  are solid.

Listing 2 shows how the OrthogonalWithNewOutput evolution is defined with these three models. As this transition only defines additions, OrthogonalWithNewOutput_before is equivalent to OrthogonalWithNewOutput_core (cf. Line 3).

**Figure:**
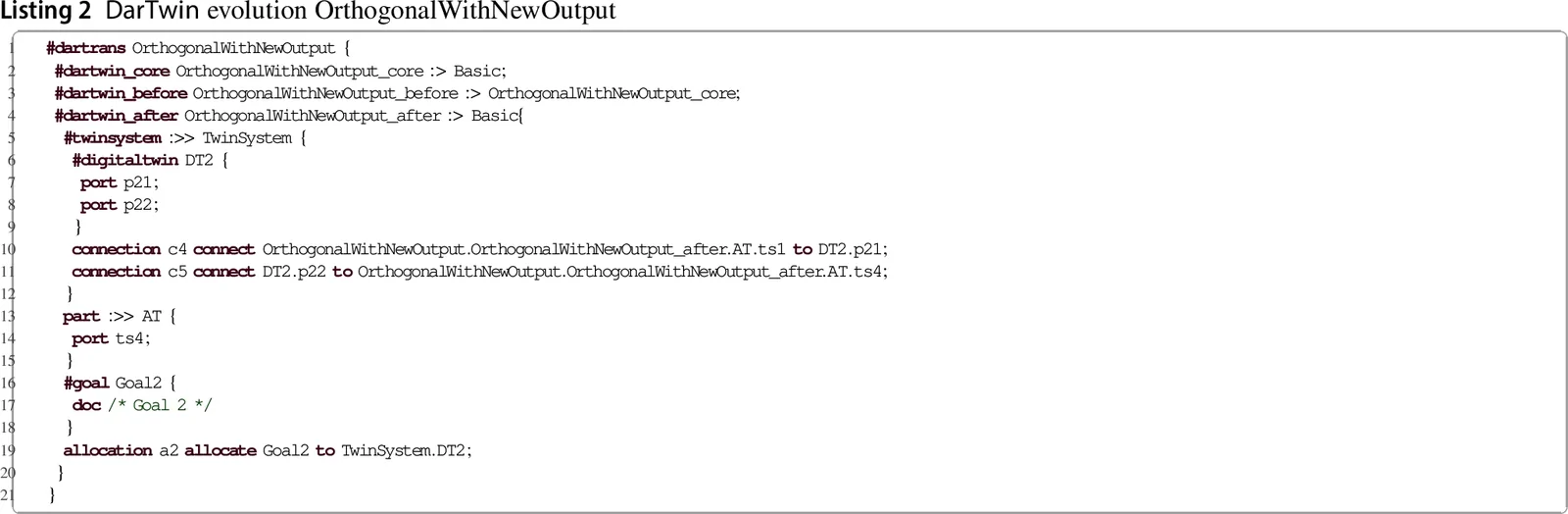

**Fig. 4:**
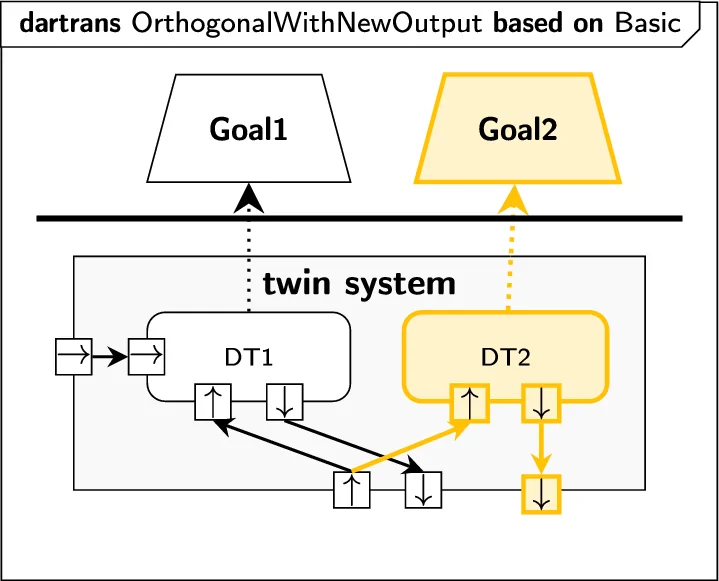
DarTrans evolution OrthogonalWithNewOutput

The graphic rendering of OrthogonalWithNewOutput is shown in Fig. 4. Orange colour indicates added elements, i.e. those described in OrthogonalWithNewOutput_after (cf. in Listing 2, Lines 4 – 20).

**Fig. 5:**
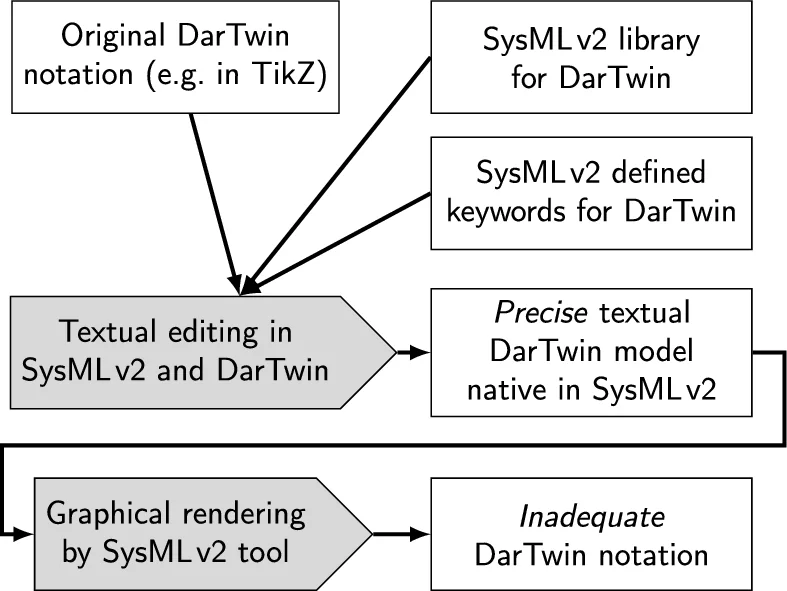
SysML v2 tooling for DarTwin DSL The rectangles describe information given to and received from the tools that are indicated in grey pentagons

#### Summarizing DarTwin defined in SysML v2

In Fig. 5 we have summarized the DarTwin tooling when using SysML v2 tools. We have included our assessments of the final DarTwin models in graphical and in textual formats. We shall clarify these assessments in the following sections.

## Defining DarTwin by MetaEdit+

Since we wanted to be able to customize our DarTwin diagrams better than what we experienced from applying SysML v2 tools, and since we still want to maintain language constraints, we decided to try and apply a dedicated DSL tool, and chose to explore MetaEdit+ since it has been used for that purpose since the 1990’s.

### MetaEdit+ basics

MetaEdit+ is a tool from the company MetaCase^2^ dedicated to supporting the design and use of domain-specific languages (DSLs).

MetaEdit+ has its own metalanguage called GOPPRR (Graph-Object-Property-Port-Role-Relationship), which in short, tells what its main concepts are. Based on their own descriptions the concepts are:

- A *Graph* is rendered as a diagram in the DSL that the user has created, and it contains instances of all the other concepts.
- An *Object* is an instance of the types that the user has created in his/her DSL and the objects may stand by itself, and may be reused in other graphs.
- A *Property* is an attribute having a value. Properties can be contained inside the other concepts of GOPPRR.
- A *Port* marks an optional specification of a specific part of an object to which a role can connect.
- A *Role* represents one connection of a relationship
- A *Relationship* represents an explicit connection between two or more objects. Relationships attach to objects via roles.

**Fig. 6:**
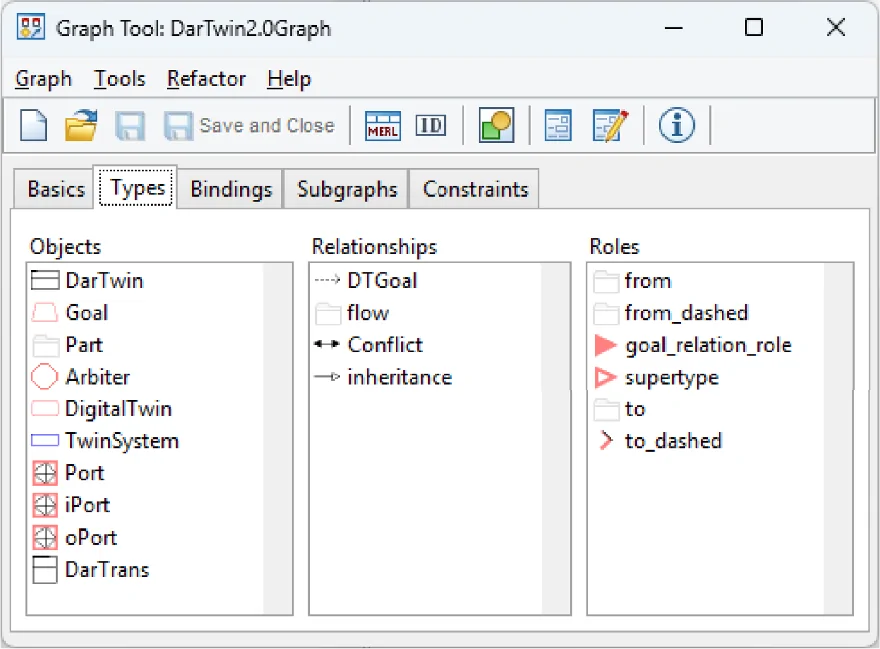
DarTwin concepts in MetaEdit+ GOPPRR

While SysML v2 defines languages with a metamodel, MetaEdit+ applies dialogues to define the concept of your DSL as shown in Fig. 6. To define the complete DarTwin it is necessary to fill out several dialogues.

**Fig. 7:**
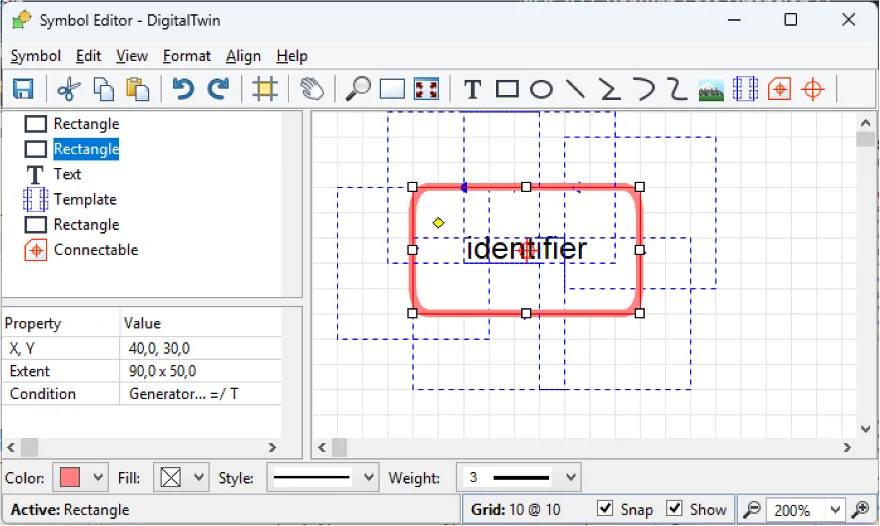
MetaEdit+ symbol editor for DarTwin DT

The types shown in Fig. 6 are associated with symbols that are placed in the graph instances. MetaEdit+ provides a symbol editor to create your own symbols as shown in Fig. 7 where we have defined our DT symbol. Notice that every graphical element of the symbol may associate a condition for when that element will be rendered in the graph based on values of the properties of the associated symbol. This is what we have applied to represent objects in orange colour and as dotted indicating new items and deleted items in the DarTrans evolution.

A DSL defined by MetaEdit+ and the graphs designed by that DSL will be stored in a *repository*. A repository can contain several *projects* but the language concepts of the projects are available throughout the whole repository. Typically, a repository is what is shared by a group of MetaEdit+ users of the same DSL.

The visibility of elements in the whole repository means that objects may be represented in several graphs. Changing an object in one graph will proliferate to all other graphs that includes that object. However, MetaEdit+ offers a way to “deep” copy elements such that they have the same properties, etc., but are still distinctly different objects. For our MetaEdit+ version of DarTwin, we apply “deep copy” because we describe the distinct elements at different times. In the description, this must be done by different descriptive elements. Typically an element will be deep copied and the copy modified to describe the element at a later stage of evolution.

### Experiences with MetaEdit+

While learning MetaEdit+ by using it, we encountered a few challenges and curiosities. It is not our intention to evaluate MetaEdit+ and compare it to other potential DSL tooling, but we recorded issues where our graphical design of the DarTwin notation was cumbersome to construct with MetaEdit+. By applying work-arounds and some simple guidelines, we consider that MetaEdit+ does provide what is necessary to construct a fruitful graphic interface.

#### Graphical challenges

- *Ports on polygons*: MetaEdit+ has an advanced rendering of ports on polygons. For DarTwin, the ports are attached to the *digital twin* and *twin system*. MetaEdit+ achieves such attachments through the associated symbols containing a *template* linked to a port concept. Ports on rounded-rectangle symbols consistently snap to the nearest edge, but the snap behaviour of a pentagon symbol used for the *Arbiter* was difficult for us to control properly.
- *Uniform scaling of* DarTwin * container elements* In the current MetaEdit+ implementation, DarTwin is represented as a single enclosing graphical symbol. When this symbol is resized, *all internal elements are uniformly scaled*, including labels, headers, and identifying metadata. If a DarTwin container is scaled down to fit a larger evolution scenario or multi-step narrative, essential textual information, such as the DarTwin name or evolution identifiers, may become partially or fully unreadable. This limitation is particularly problematic for DarTrans diagrams, where the DarTwin identifier carries semantic meaning (eg., core/before/after). Since text scaling cannot be selectively controlled, preserving readability requires manual layout compromises that limit diagram density.
- *Graphical constraints*: While MetaEdit+ has options for constraining the topography of the diagrams, it does not seem possible to constrain how objects are placed geometrically. For DarTwin we would have liked to constrain DTs at the lower part of the surrounding frame, and the goals above the horizontal dividing line. As an alternative to a syntactic constraint, we have integrated into the SysML v2 generator checks for such graphical containment. No SysML v2 is generated if the containment is broken.
- *Text search and replace* Even for graphical editors it is very useful to be able to find elements by text. For MetaEdit+ that would also be very useful, but we did not find any such search mechanism.

#### Conceptual challenges

- *The scope of an object*: when an object is created, it is visible and changeable throughout the entire repository. For DarTwin describing evolutions, it is desirable to keep several snapshots of the systems related to the same narrative and development. Therefore, we had to make “deep copies” of graph content. This would mean several objects with the same properties in the same project. Failure to perform deep copying would cause modifications to propagate unexpectedly to diagrams representing earlier or later system states.
- *Nesting*: While MetaEdit+ has a hierarchy of concepts representing containment, containment is not a core concept itself. We cannot easily express that twin systems contain DTs. We can define a relation representing this containment, but relationships are normally represented by lines, which would quickly make diagrams less readable. There is a concept of a “subgraph”, but it introduces a new level of diagram that would not be our intention.

#### Code generation

We implemented a validation generator to enforce the intended structural conventions of DarTwin diagrams prior to code generation. The validation checks that all goals are placed within the enclosing  container and above the horizontal divider, that each  is contained within its corresponding , and that the  itself is positioned below the divider and within the bounds of the  frame. SysML v2 code is generated only if these constraints are satisfied, thereby making graphical well-formedness an explicit precondition for semantic translation.

Goals could be generated more directly from their model properties (Purpose and an optional goal explanation), while inheritance was detected through explicit goal-to-goal generalization links.

Although a generator is used to translate DarTwin models defined in MetaEdit+ into SysML v2, this integration is strictly one-directional. The generated SysML v2 models are intended to support further detailing and inspection, but changes made at the SysML v2 level cannot be propagated back to the MetaEdit+ DarTwin models.

### Summarizing DarTwin defined with MetaEdit+

**Fig. 8:**
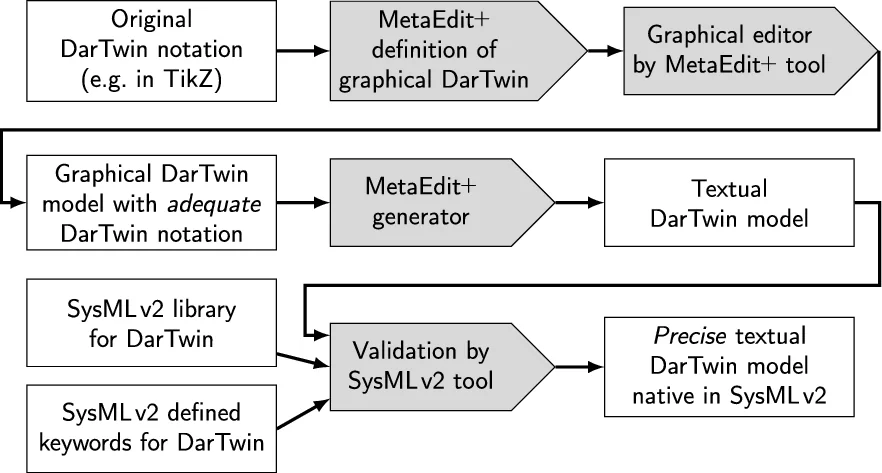
DarTwin tooling based on MetaEdit+ and SysML v2. The rectangles describe information given to and received from the tools that are indicated in grey pentagons

In Fig. 8 we summarize our DarTwin tooling that applies both SysML v2 and MetaEdit+ tools. We see that our tooling is more complex than with only SysML v2, but this was necessary to mitigate the graphical rendering issues.

## DarTwin tooling on MetaEdit+ compared with SysML v2 tooling

**Fig. 9:**
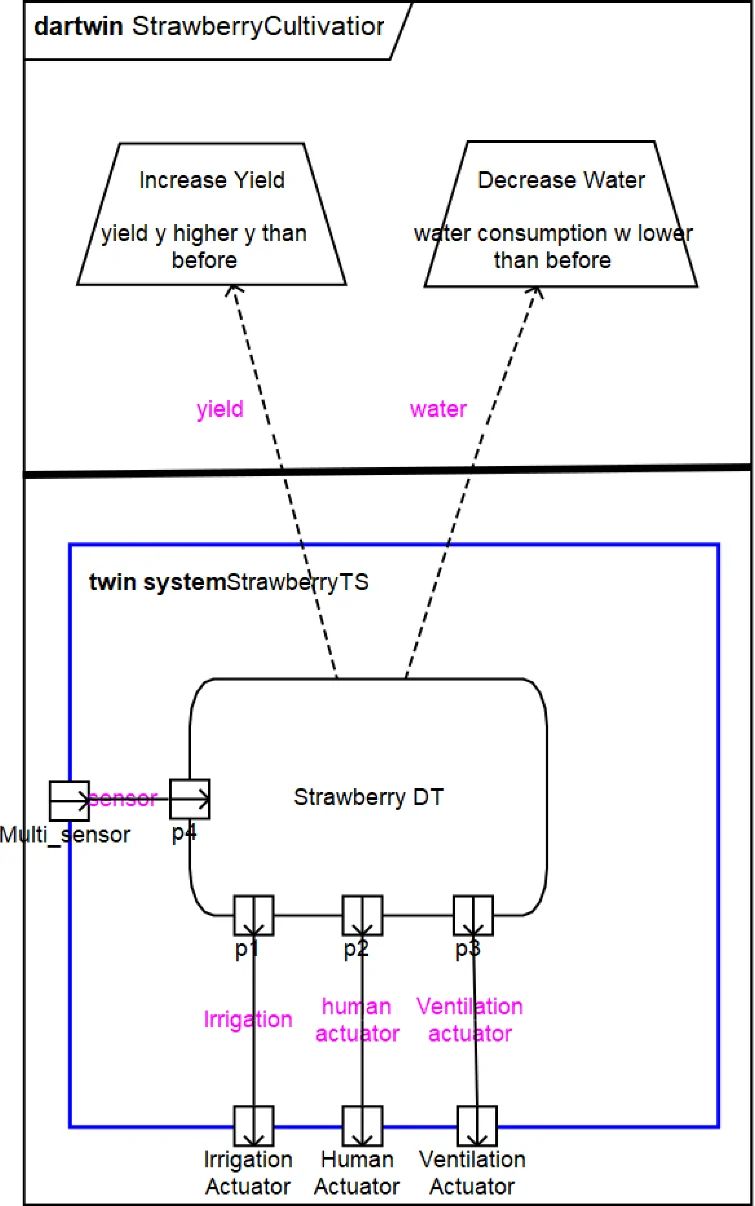
Strawberry cultivation system DarTwin in MetaEdit+

**Fig. 10:**
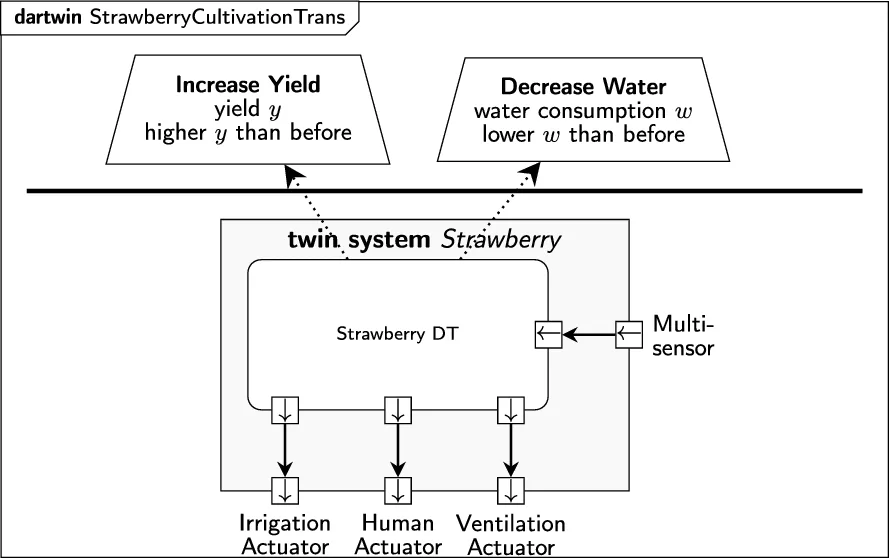
Manually edited DarTwin representation of the strawberry cultivation system.

In this section we shall revisit our findings from [24] where we showed how three different SysML v2 tools rendered DarTwin. Compared with our DarTwin notation rendered by Tikz (Fig. 10), they clearly showed that the intuitive overview expressed through the notation rendered by Tikz could not be properly achieved through any of the SysML v2 tools that we tried. In Fig. 9, we show how MetaEdit+ renders the same Strawberry example.

SysML v2 tooling provides semantic precision and standards alignment, but cannot still provide the graphical rendering with necessary similarity to our DarTwin notation. MetaEdit+ provides full control over language and notation, but we need to program generators to preserve the connection with our textual DarTwin in SysML v2 syntax.

As a means of checking the completeness and applicability of the DarTwin DSL, we look at two case studies that feature evolution. In this section, we show how the strawberry cultivation system can be graphically presented by different tools, and in Sect. 5, we show how a gantry crane system [36] is used to demonstrate systematic evolutions.

### SysML v2 tooling for DarTwin

To evaluate the applicability of DarTwin DSL within existing SysML v2-based tooling environments, we modeled a moderately complex DT system for indoor strawberry cultivation. The system was selected as a representative validation use case since it represents a real case including sensors, actuators and human interaction which can be described by DarTwin in a fairly simple diagram.

The modeled DT supports environmental monitoring, irrigation and ventilation control, and operator assistance through alerts and operational feedback. The desired results for these functionalities were represented through explicitly defined goals and realized using a composite twin structure, illustrated in the manually arranged DarTwin rendering shown in Fig. 10. The manual adjustments performed in this figure were limited to visual arrangement and styling in order to approximate the intended DarTwin notation and did not alter the underlying model semantics.

**Fig. 11:**
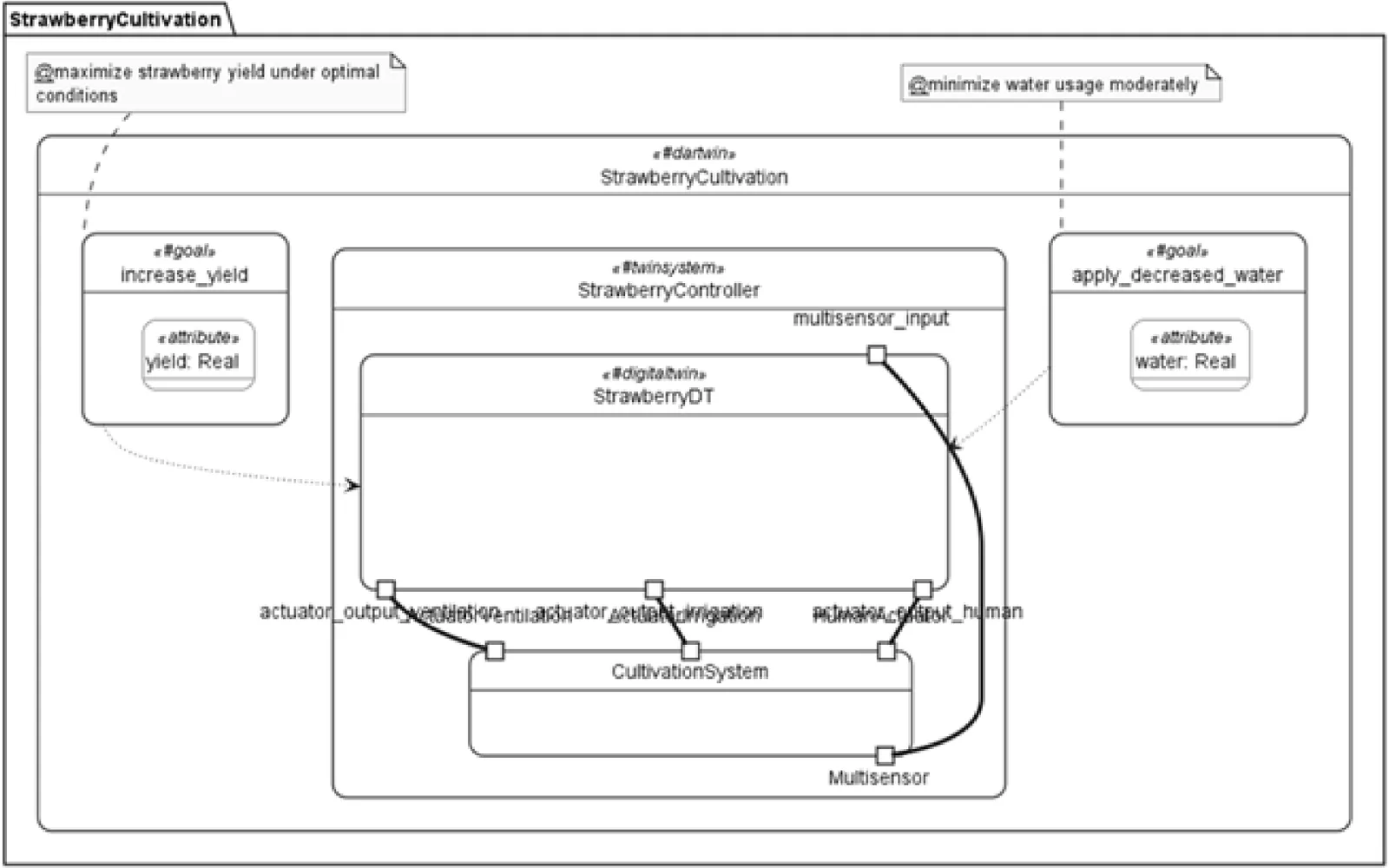
This rendering was produced using the pilot implementation.

**Fig. 12:**
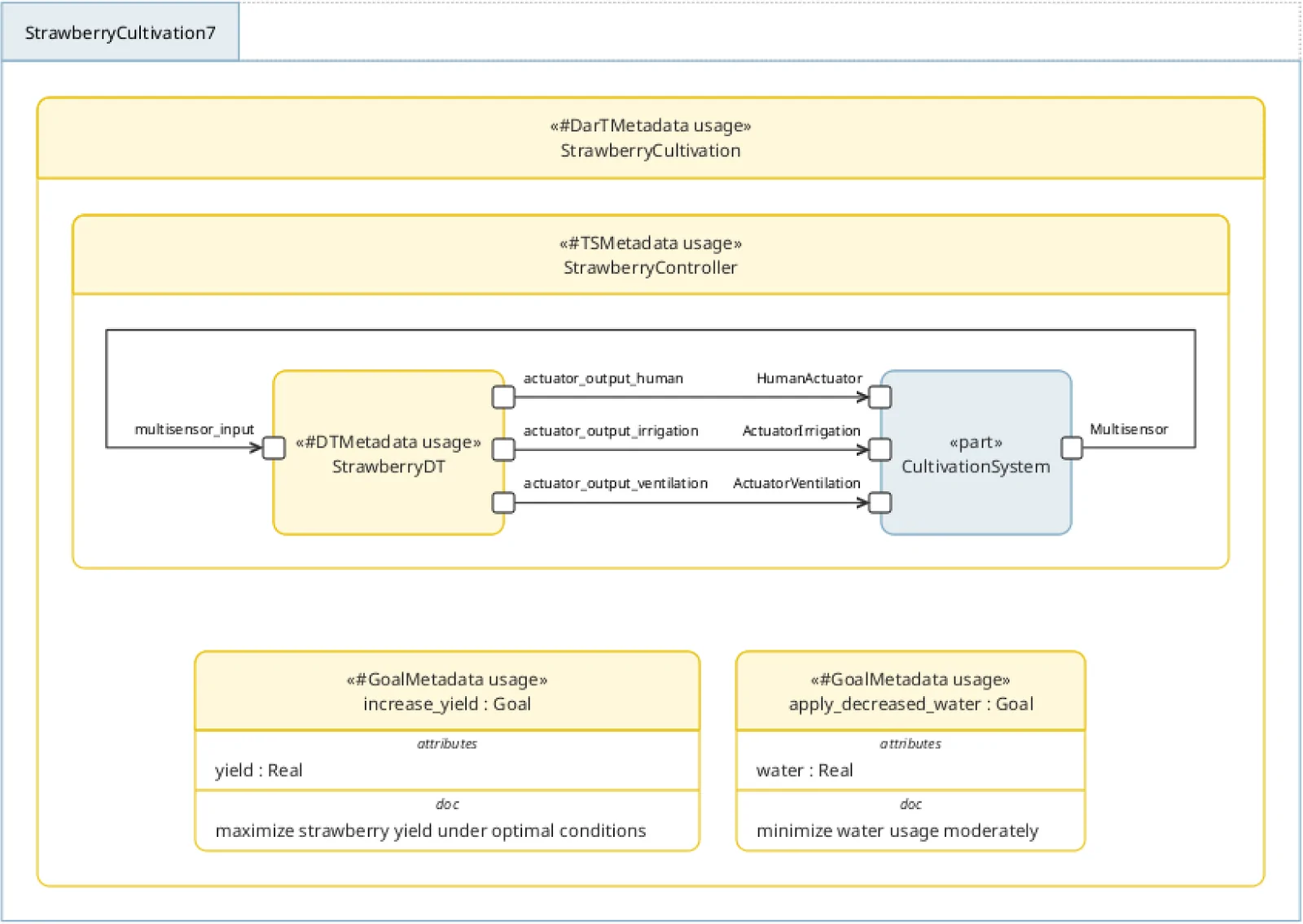
This rendering was produced using Tom Sawyer SysML Viewer v1.3.

**Fig. 13:**
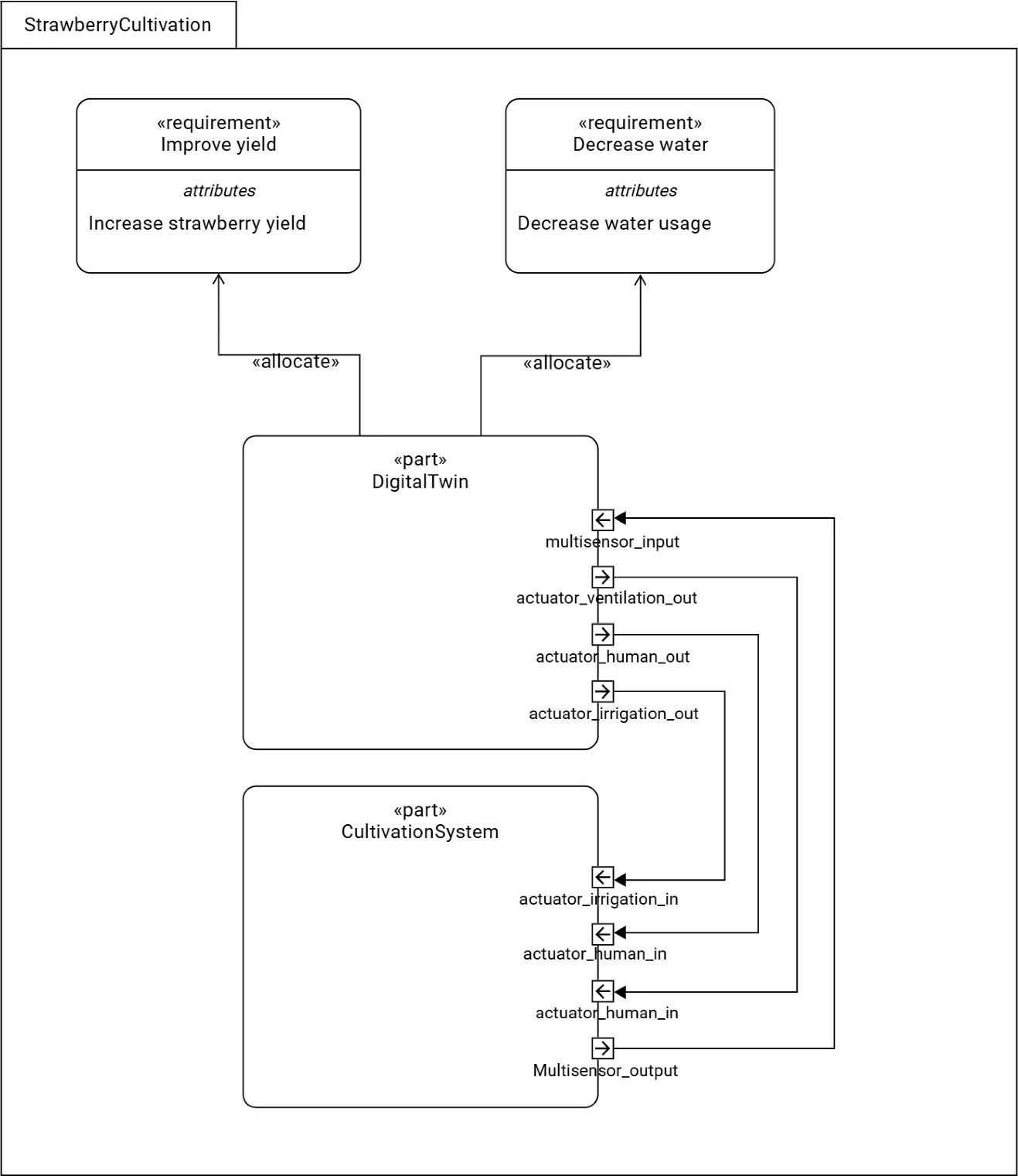
This rendering was produced using SysOn 2026.1 with manual visual style.

The model was defined textually using a single  block containing one , one  element, structural relationships defined through , and goal allocations specified using  as shown in Listing 3. The same textual model was subsequently rendered using multiple SysML v2-compatible tooling environments. Figure 11 shows the rendering produced using the SysML v2 Pilot Implementation [41], Fig. 12 presents the rendering generated using Tom Sawyer SysML Viewer [52], and Fig. 13 shows the corresponding SysON rendering [13].

The evaluation focused on three aspects: (1) syntactic acceptance of the textual DarTwin DSL model, (2) semantic completeness of the generated graphical representations, and (3) preservation of the intended DarTwin visual conventions proposed in [36].

**Figure:**
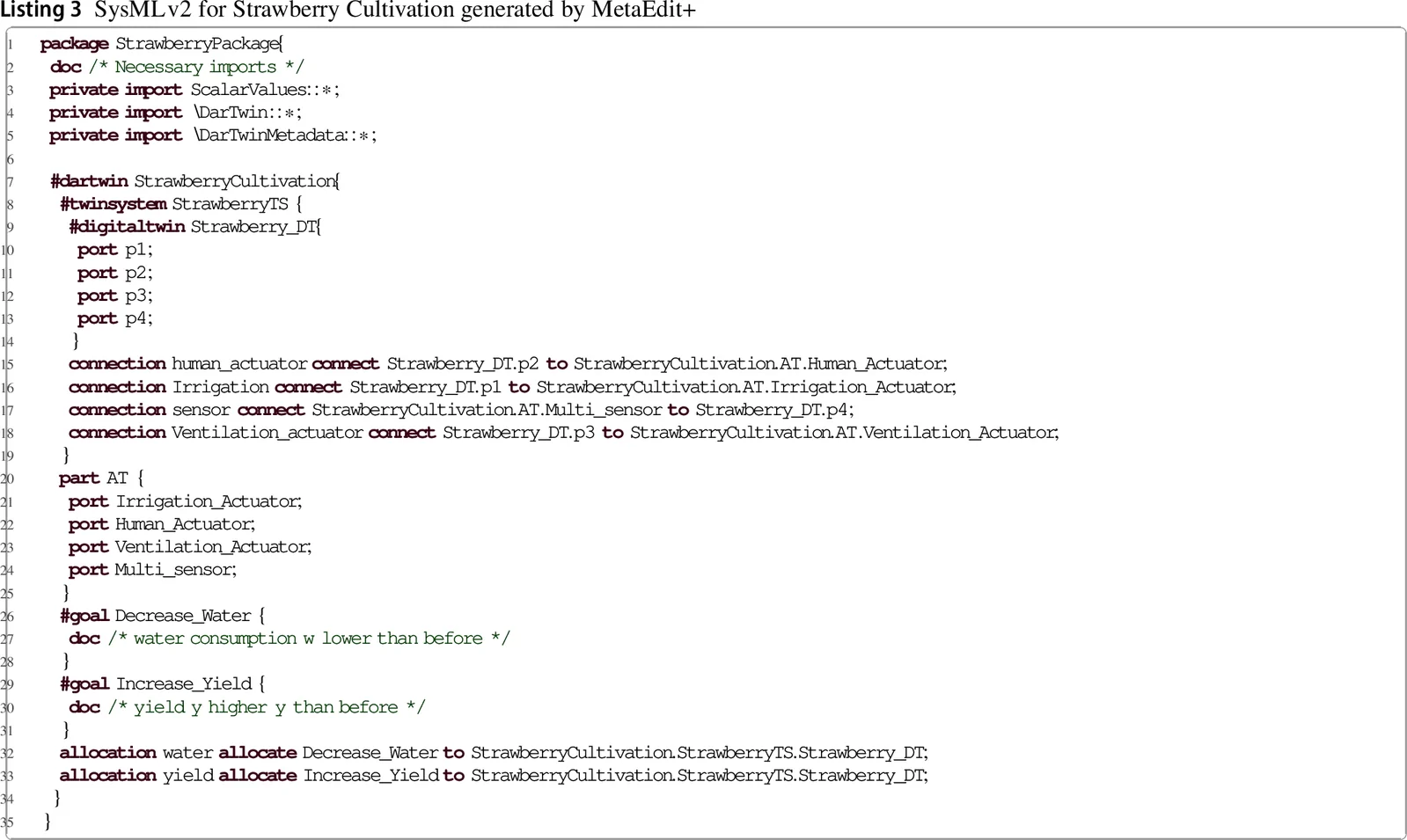

Although all evaluated tools successfully accepted the textual DarTwin DSL model, none of them were capable of reproducing the intended DarTwin visual notation without substantial manual intervention.

- **SysML v2 pilot implementation (eclipse plugin 2026.01)** accepted and parsed the textual model successfully. However, graphical rendering relied on a fixed and outdated PlantUML^3^ back-end with limited layout control. The resulting diagrams contained excessive white space, poor edge routing, and misplaced elements. In particular,  blocks were rendered outside their associated  containers. The rendered diagram is shown in Fig. 11.
- **Tom Sawyer SysML viewer (v1.3)** is based on the Pilot Implementation and provided improved graphical flexibility compared to the Pilot Implementation, but auto-layout behaviour was non-deterministic across refreshes. Manual clean-up was required to remove unnecessary metadata and reorganize the diagram structure. Key DarTwin visual conventions, such as hierarchical goal placement and horizontally aligned twin structures, could not be enforced automatically. The rendered diagram is shown in Fig. 12.
- **SysON (v2026.1)** accepted the textual syntax but failed to render several semantically valid model elements correctly. Goals, requirements, and complex connection statements were omitted or rendered inconsistently, even when port paths were fully qualified. The generated diagrams contained anonymous parts, incomplete structures, and no meaningful diagnostic feedback. Since SysON does not recognize DarTwin DSL-specific extensions such as , the resulting graphical representation could not be customized for DarTwin-oriented visualization. The rendered diagram is shown in Fig. 13.

Based on our experience with the Strawberry Cultivation System, we observed that the DarTwin DSL metamodel can successfully capture structural relationships and goal allocations within DT systems at the textual level. At the same time, the work with this use case exposed limitations in current SysML v2 tooling support for preserving domain-specific visual semantics and notation conventions associated with DarTwin modelling.

These limitations motivated the exploration of alternative approaches for graphical rendering and editing support while still maintaining compatibility with the underlying SysML v2 representation.

### MetaEdit+ tooling for DarTwin

Our primary motivation for introducing MetaEdit+ was to improve graphical editing and rendering support for DarTwin beyond what was available in existing SysML v2 tooling. As illustrated in Fig. 9, the resulting notation is visually closer to the intended DarTwin representation shown in Fig. 10.

Although the resulting diagrams resemble the intended DarTwin notation and therefore provide the graphical intuition we wanted, several practical and conceptual challenges remained.

Since our DarTwin diagrams are rather simple in order to make them fit reasonably well into our presentation, creating them with MetaEdit+ from scratch is not very complicated once you get used to the tool. The “deep copy” mechanism is essential when dealing with evolutions, since modifying shared elements across transition states may otherwise unintentionally affect earlier or later configurations. Still, MetaEdit+ makes the creation of DarTwin diagrams sufficiently easy.

The difference between the SysML v2 language and the MetaEdit+ way to define a DSL means that the syntax is less controlled in MetaEdit+ than we would like. In particular, this relates to nesting and graphical placements. We have mitigated this shortcoming by including in the SysML v2 generators a test on our graphical constraints. If e.g. not all DTs are inside the twin system, an error report is given, and no SysML v2 is produced.

Compared to the evaluated SysML v2 tooling, MetaEdit+ provided substantially improved control over graphical rendering and notation layout. MetaEdit+ was meant to mitigate the shortcomings of graphical presentation and editing, while semantic alignment is preserved through generators producing textual SysML v2 representations from the graphical models. We want to maintain our relationship between DarTwin and SysML v2 and continue to rely on the SysML v2 semantics, as well as the potential transition from DarTwin to SysML v2 development in concrete projects. Therefore, we have made generators for producing SysML v2 from DarTwin and DarTrans graphs.

**Fig. 14:**
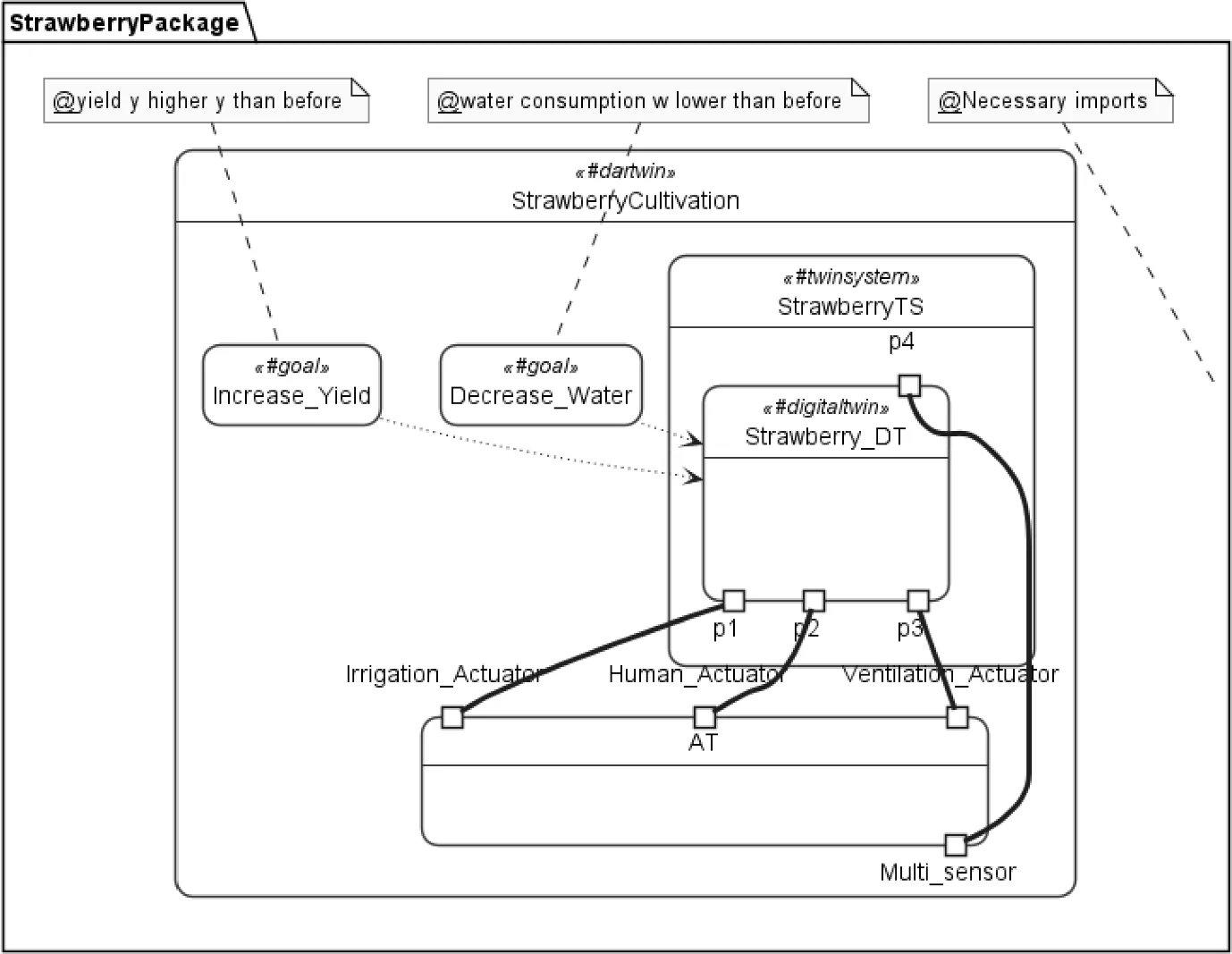
Strawberry cultivation system from MetaEdit+ via SysML v2 rendered by Pilot Implementation

Just for comparison with Fig. 11 the generated SysML v2 rendered by the Pilot Implementation in Fig. 14.

## DarTwin methodology performed with MetaEdit+

In this section, we present our DarTwin methodology for applying generic evolution pattern to generate a concrete DarTrans transition. In Sect. 5.1, we repeat and extend from [24] how textual SysML v2 can be used to systematically construct the structure of the concrete DarTrans. Then, in Sect. 5.2, we show how applying our new tooling on MetaEdit+ would be performed. The question is whether using graphics with MetaEdit+ has advantages over applying textual DarTwin on SysML v2.

### Applying transition patterns with SysML v2

**Fig. 15:**
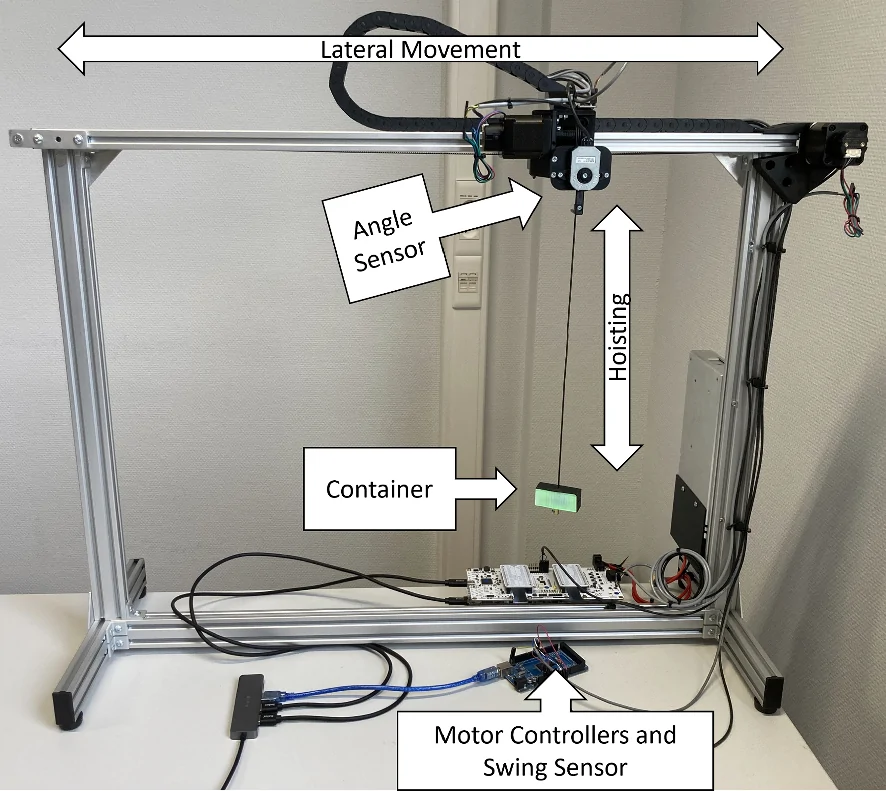
The scaled gantry crane

The University of Antwerp’s Cosys-Lab hosts a lab-scale gantry crane case study, which serves as a case study platform for research into DT systems. The crane is depicted in Fig. 15 and models the behaviour of the gantry cranes operated by Antwerp harbour, where full-scale gantry cranes are employed to transfer containers between docked vessels and the quayside. This case study’s implementation details can be found in [35]. Building upon this foundation, [36] introduced a series of case study evolutions designed to illustrate transition workflows in the development of a crane DT system. In the present work, one such evolution is revisited as a vehicle for demonstrating .

The systematic evolution of a target system via a  evolution template is governed by a “*5-step procedure*”, which is illustrated here through an example drawn from the gantry crane system. Specifically, the objective is to upgrade the DT responsible for trajectory generation by substituting a Linear Quadratic Regulator (LQR) with an Optimal Control Problem (OCP) solver. This is achieved by applying the generic Replacement transition template, defined in Listing 4, which facilitates the substitution of one DT for another whilst reallocating the goal originally assigned to the former to the latter.

**Figure:**
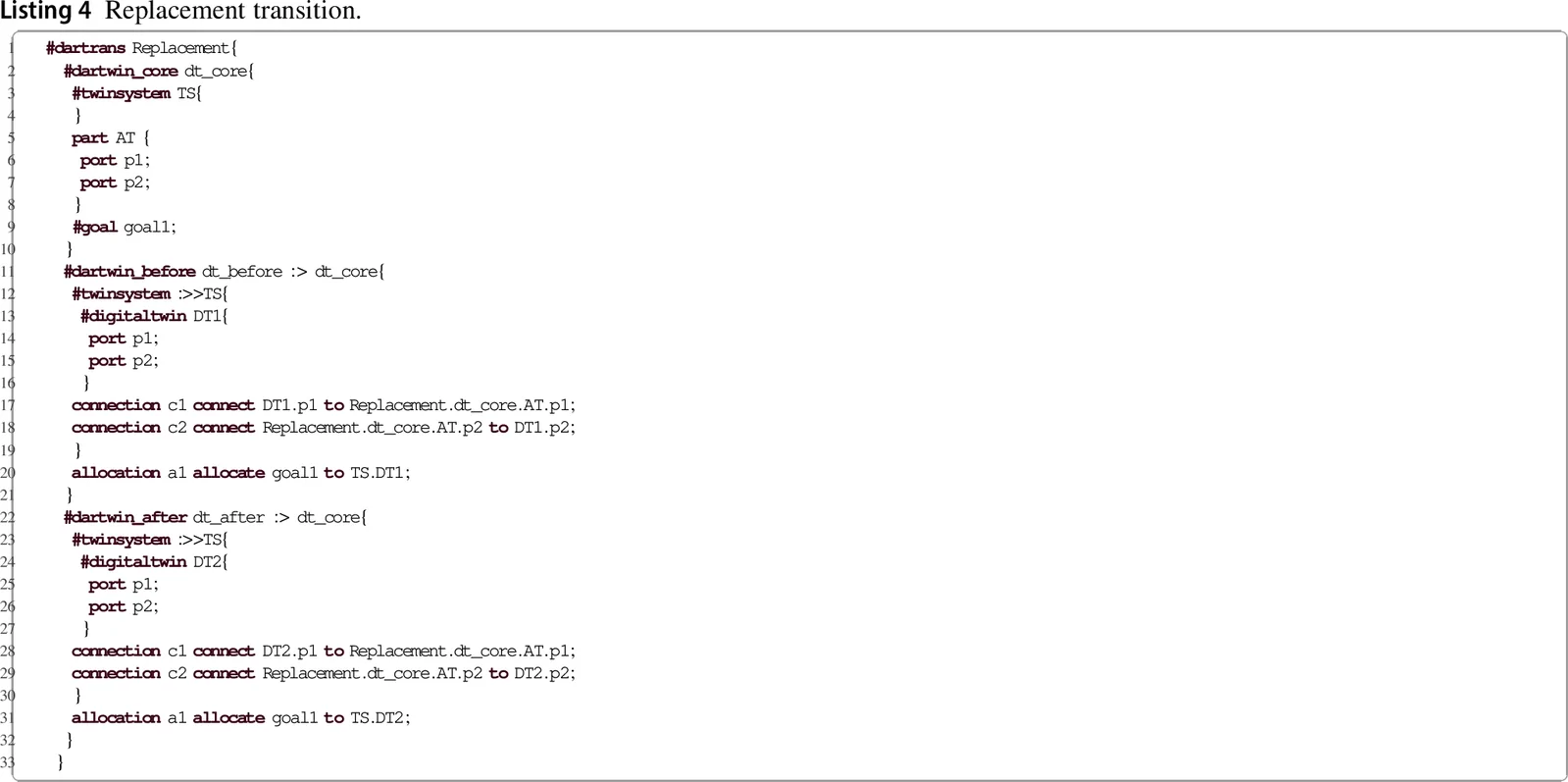

**Fig. 16:**
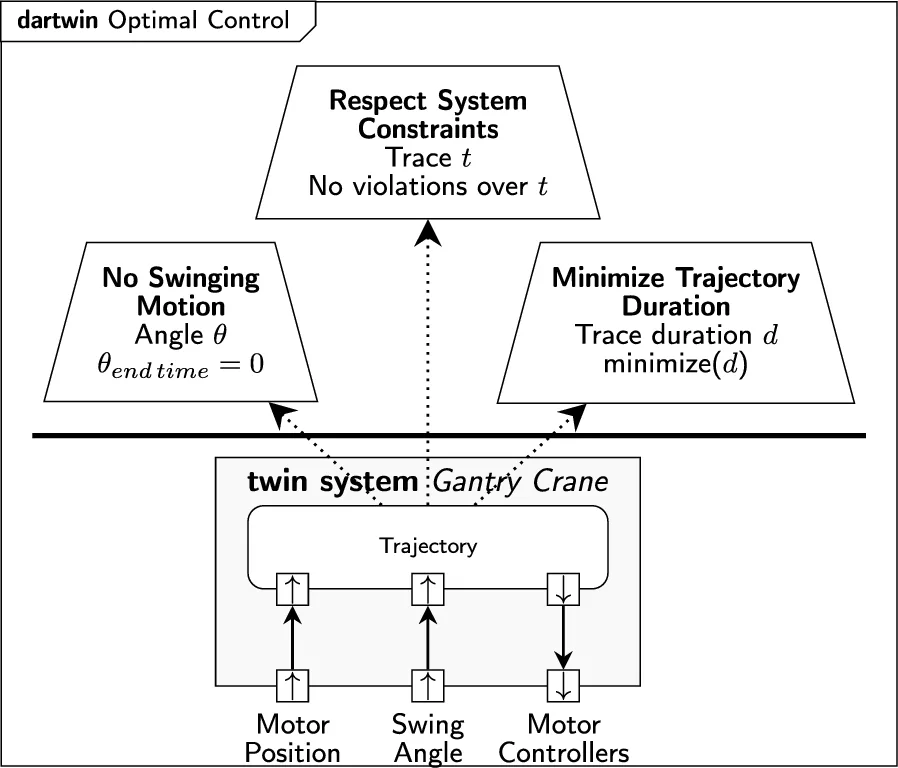
Optimal Control DarTwin of the Gantry Crane system

The role of a generic transition pattern (like Replacement) is to be able to apply a given well-understood strategy for evolution instead of having to develop an evolution strategy from scratch. However, having chosen a generic transition, the method still will need supplements from the target domain to connect the generic pattern to the target DT system. First we must make sure the target DT system really can apply the generic pattern. This means that the pattern starting point must match (be an abstraction of) the target DT system starting point. Then the generic elements describing what needs to be removed are marked on the target DT system. These changes are all done manually by the DarTwin user. Then there is the need to supplement generic elements that should be added with corresponding ones for the target DT system. This is done by inheritance. Finally the whole system is flattened meaning that the target elements are merged with those from the generic pattern they inherit. Those target elements that are marked to be removed are deleted. The final result is a plain DarTwin DT system. We shall now go through one evolution of the Gantry DT system based on the generic Replacement transition.

A transition definition comprises three constituent models: the , which encapsulates the AT alongside its ports and the associated goal – elements that remain invariant throughout the transition; the , which specifies the original DT together with its ports, connections, and goal allocation; and the , which defines the replacement DT with its corresponding ports, connections, and goal allocations.

The application of this transition to the gantry crane example system is presented in Listing 5. It should be noted that the system is, in its complete form, considerably more complex, as illustrated in Fig. 16; for the sake of conciseness, the code listings have been simplified to a single goal and two ports, rather than the full complement of three of each.

**Figure:**
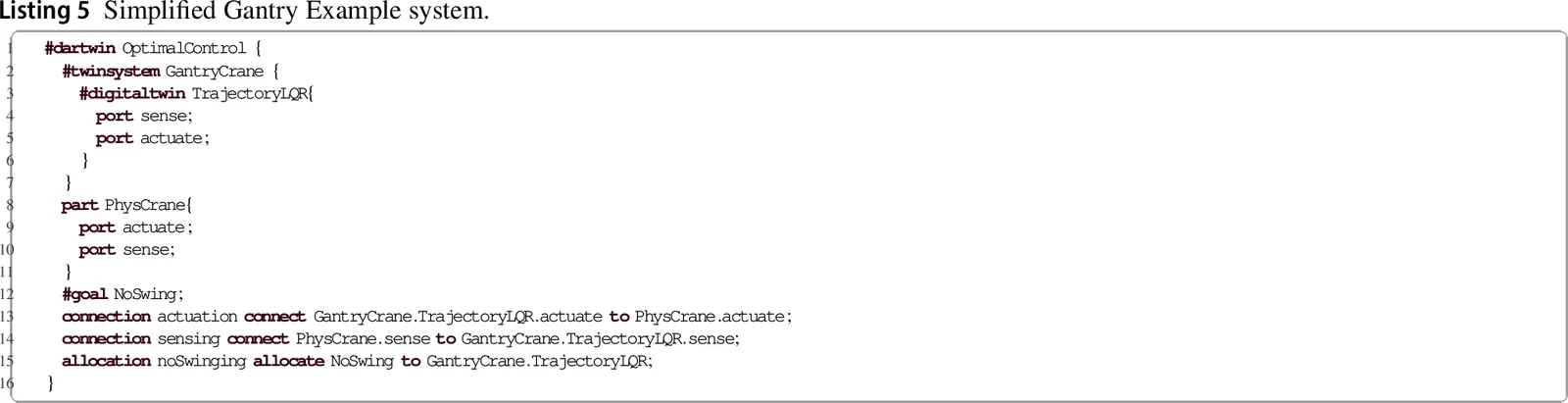

**Fig. 17:**
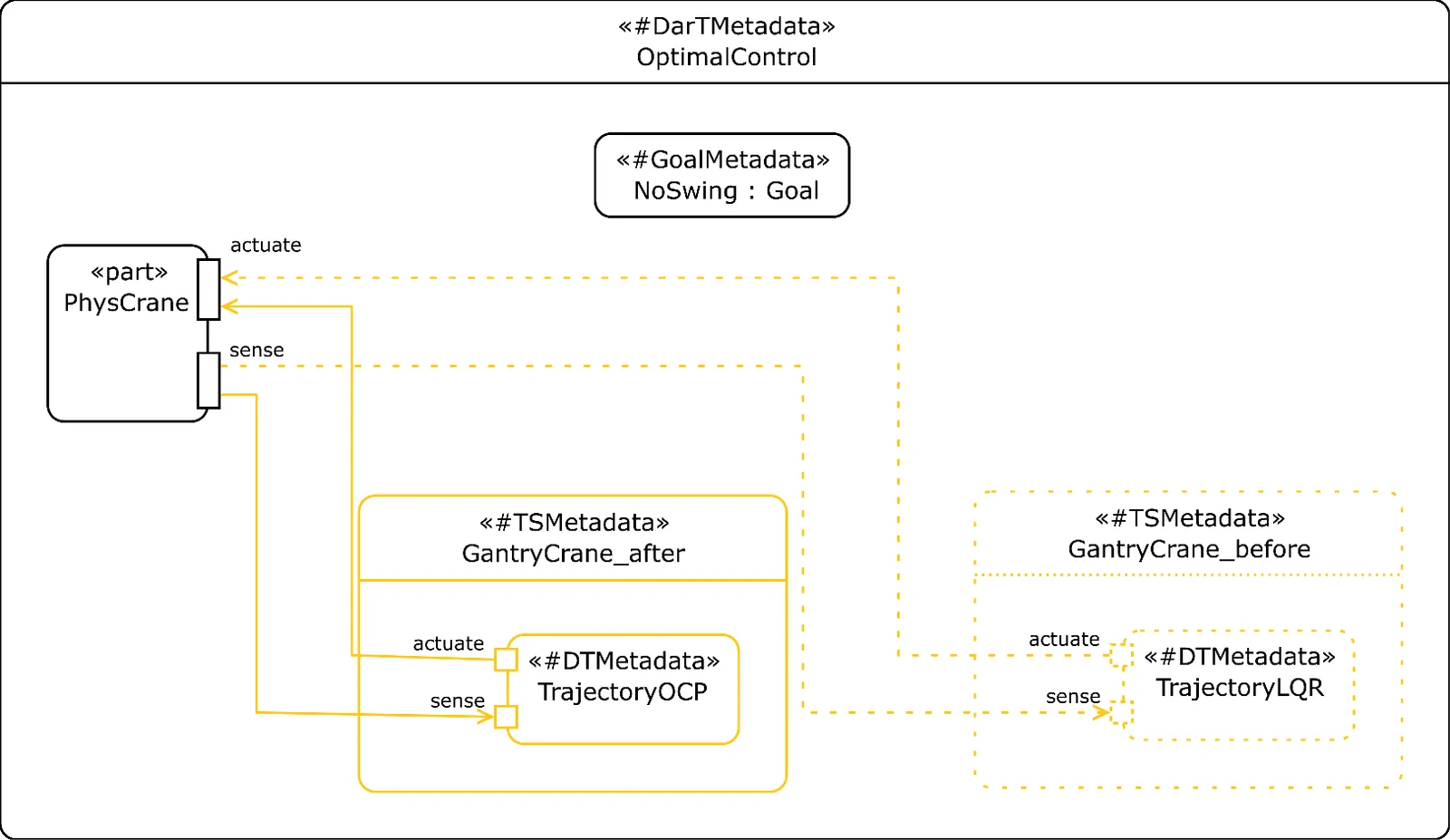
Visualization of the replacement transition on the example made with the Tom Sawyer SysML v2 Viewer tool. Elements are highlighted as follows: deletions are dashed orange, additions are solid orange, and unchanged elements are solid black

Next, we apply the 5-step procedure.

1. We take the gantry system from Listing 5 as is as input.
2. We take the replacement pattern (Listing 4), and make sure our OptimalControl example can specialize all the elements in  and also including those in inherited . of Replacement. This defines the premise: *Can the chosen transition pattern be applied?* This is shown in Listing 6.

**Figure:**
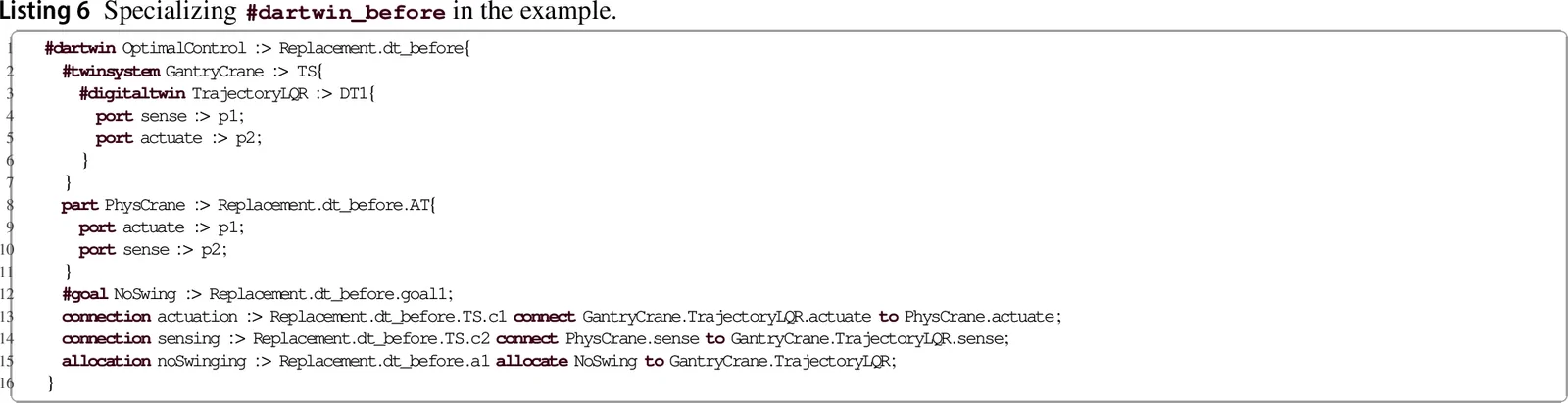

3. We delete all elements that are part of the  of Replacement such that only elements of the  remain. The result is shown in Listing 7.

**Figure:**
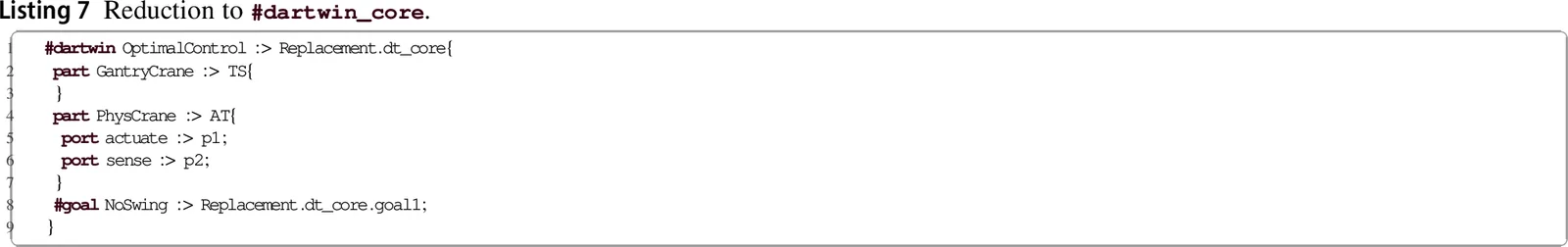

4. We add all the elements of the  to the example system by specializing it instead of . The additions of the  of the pattern must also be themselves specialized to add system-specific properties. This is shown in Listing 8.

**Figure:**
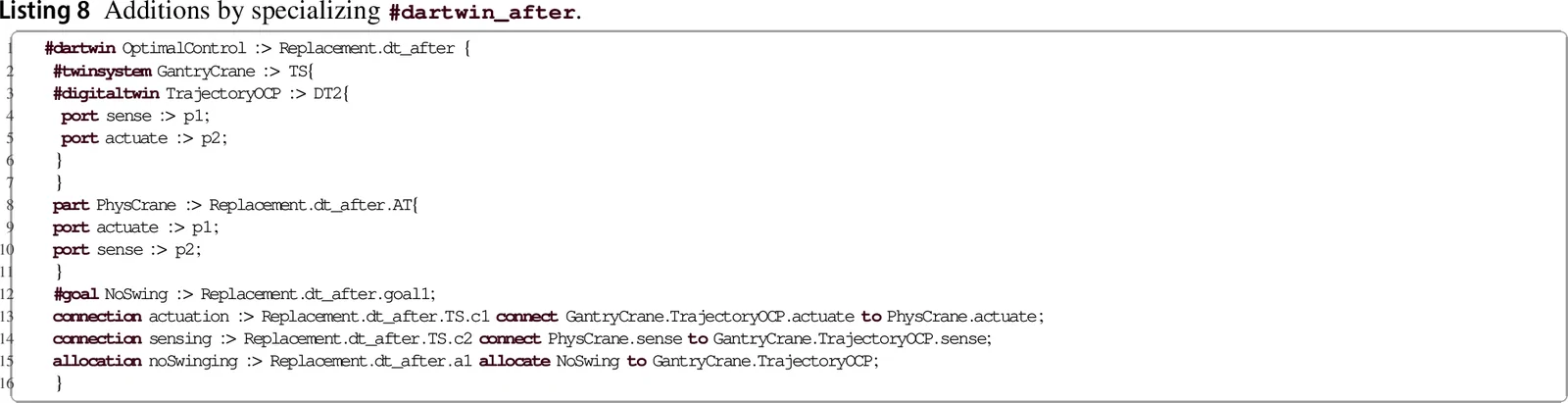

5. We want to merge the inherited  of the pattern with the transformed system to get a plain  with no mentioning of the pattern. Since the pattern we have here is very general and defines only structure, we may in fact just remove all references (inheritances/specializations) to the pattern to finalize the transition. This is shown in Listing 9.

**Figure:**
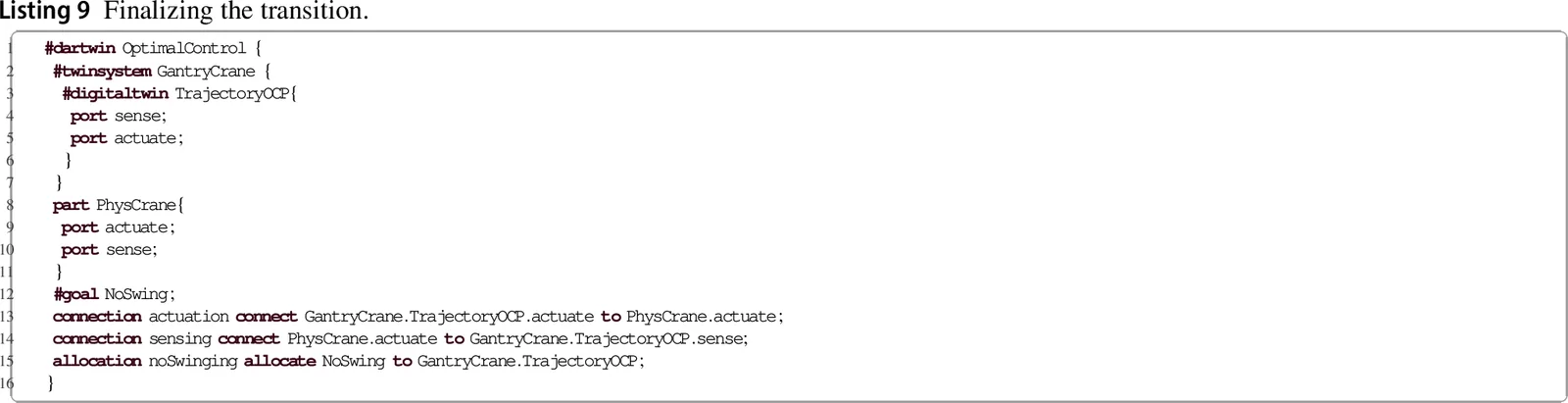

Upon completion of this sequence, the replacement transition is complete, and the optimal control  has been successfully updated. To provide a visual overview of the transition Fig. 17 presents an annotated summary of the changes applied to the example system, wherein modified elements are highlighted in orange and unchanged elements are rendered in black. It should be noted that the visualization was generated by exporting to SVG from the Tom Sawyer SysML v2 Viewer tool; as a consequence, certain connections, such as linking the goal to the DTs, were absent from the figure. Furthermore, colour annotations were subsequently applied using an SVG editor. The amount of post-editing necessary to achieve this rendering is beyond what a user would like to perform every time the modelling tool is used.

**Fig. 18:**
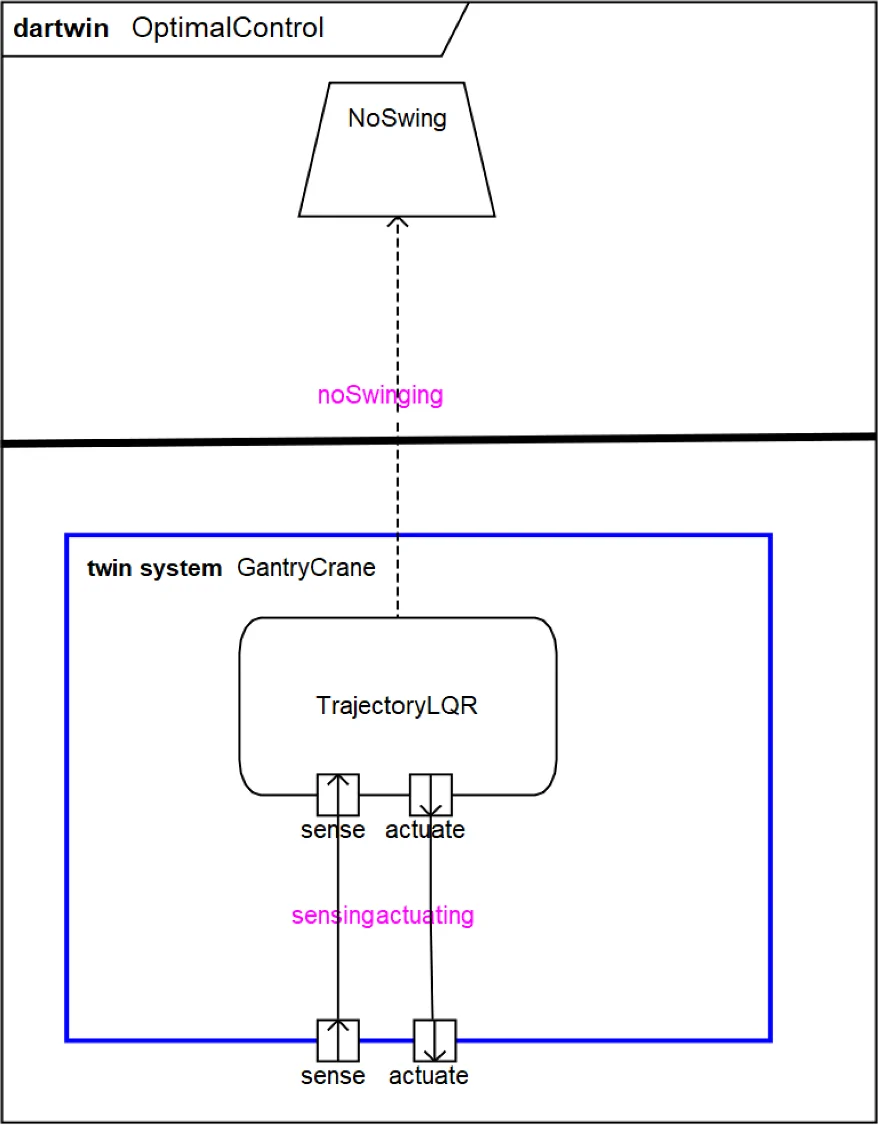
Optimal control in MetaEdit+

The primary observation from applying these and other transition examples is that in the original publication [36], the transitions were interpreted in a rather liberal and open way. Their formalization within DarTwin DSL has brought this to light, whilst simultaneously clarifying the requirements for a rigorous and well-formed definition of  to support a systematic DarTrans method for evolution.

### Applying transitions with MetaEdit+

The DarTwin approach to apply general transition patterns using SysML v2 consists of a series of specializations using a textual notation, redefining and flattening. Using MetaEdit+ would mean applying graphics rather than text, and our hope was that it would be perceived to be more intuitive on each step. DarTrans evolutions rendered in MetaEdit+ apply orange colour to identify evolutionary changes like our original DarTwin notation.

Our starting point is the simplified version of the Gantry as shown in SysML v2 in Listing 5. In MetaEdit+, this is shown in Fig. 18.

**Fig. 19:**
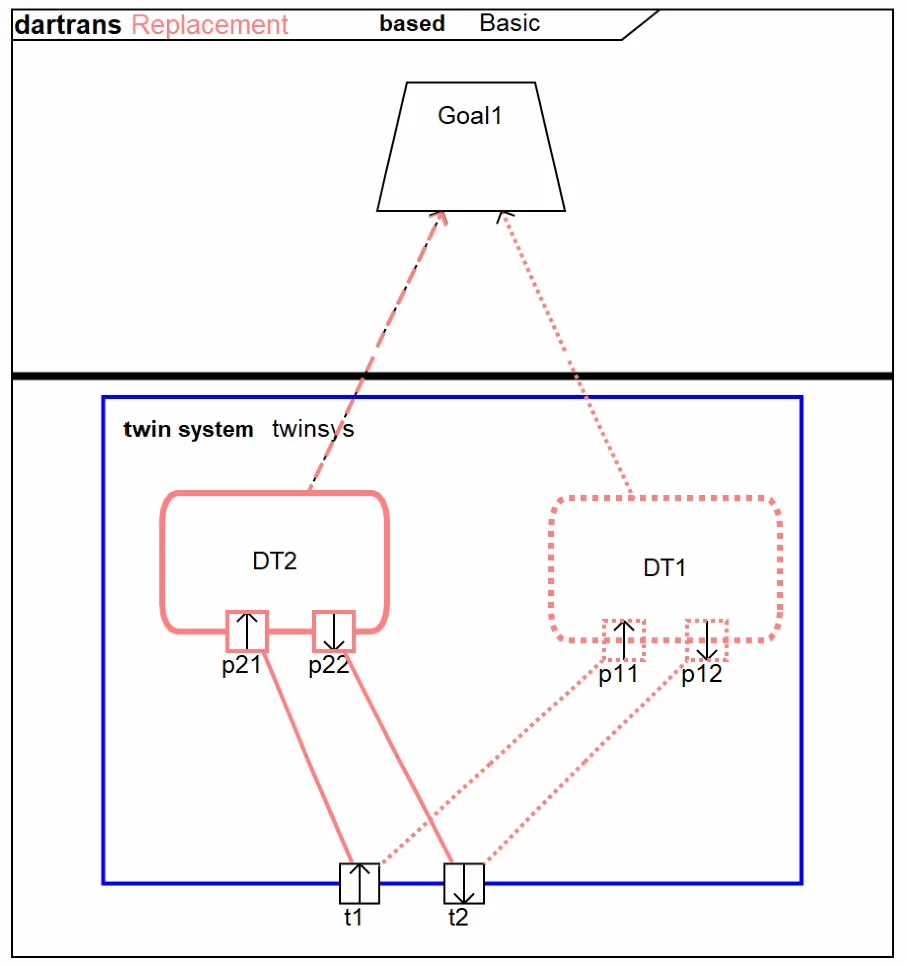
Replacement DarTrans in MetaEdit+

Our *trigger* for wanting to evolve is that we have found a better algorithm and the *goal* would be to make the crane swing even less. We believe that the existing DarTrans Replacement (Fig. 19) should apply.

**Fig. 20:**
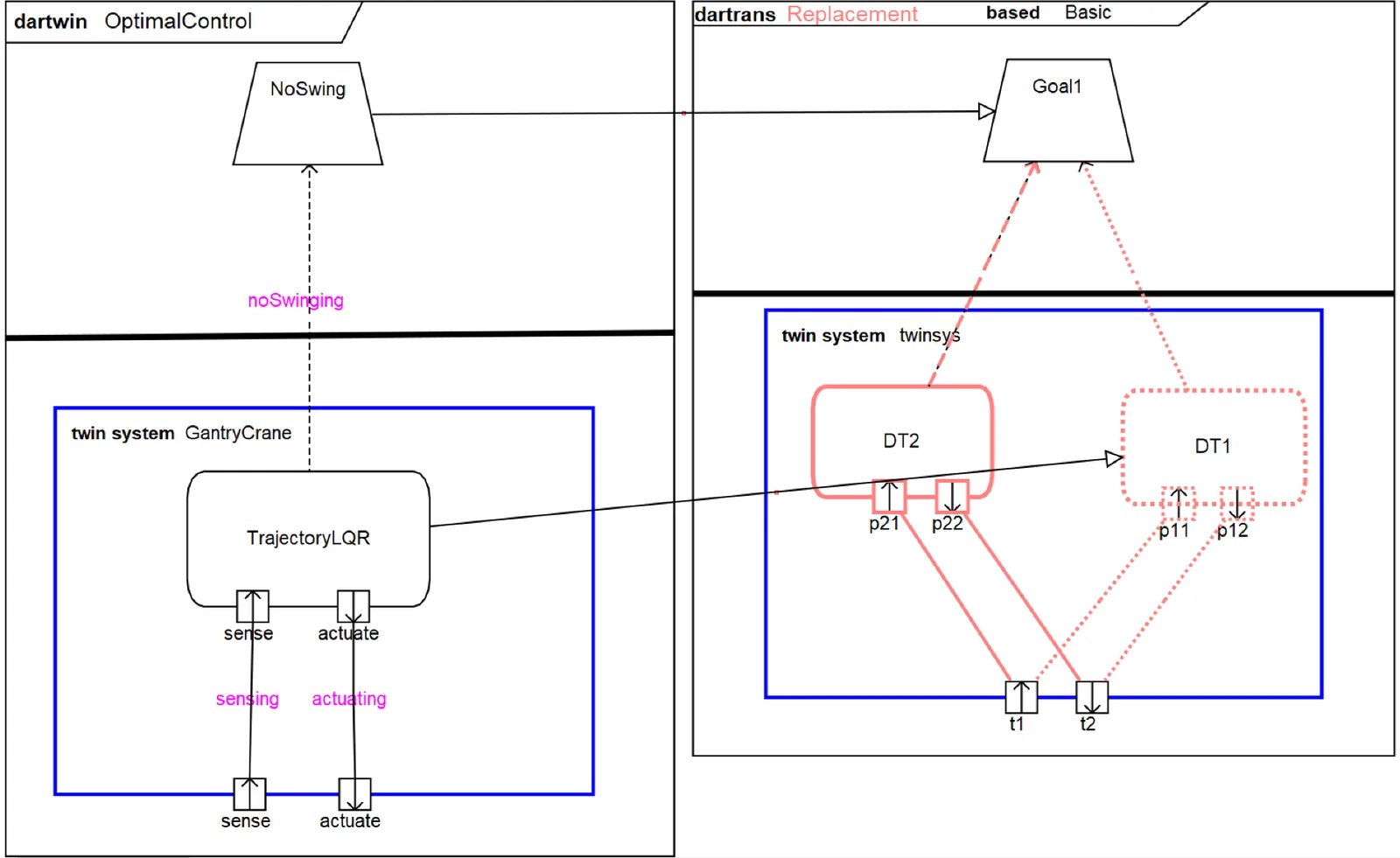
Can the DarTrans *Replacement* be applied?

The question now is whether we are sure that the chosen pattern does fit. We place the DarTwin OptimalControl and the DarTrans Replacement side-by-side and apply inheritance on the major elements to indicate what elements of the actual system should match the starting point of the DarTrans as shown in Fig. 20. In a DarTrans, the elements to match are all those that are black or orange dotted which together defines the .

We also need to check that the ports match properly. The concrete system may have DTs with more ports than those shown in the evolution DarTrans, but all those of the pattern need to be logically inheritable in the concrete DT (and twin system). Here we find that the *Replacement* should fit.

Now we want to merge the starting point of the generic DarTrans (Replacement) and its corresponding elements of the  concrete system (OptimalControl). There is no reason to do anything with the concrete black elements, but such merger means to transfer the deletion instructions of the DarTrans onto the corresponding elements. In MetaEdit+, we have indicated “to be deleted” just by a Boolean property that has the visual effect that the symbol is orange dotted.

**Fig. 21:**
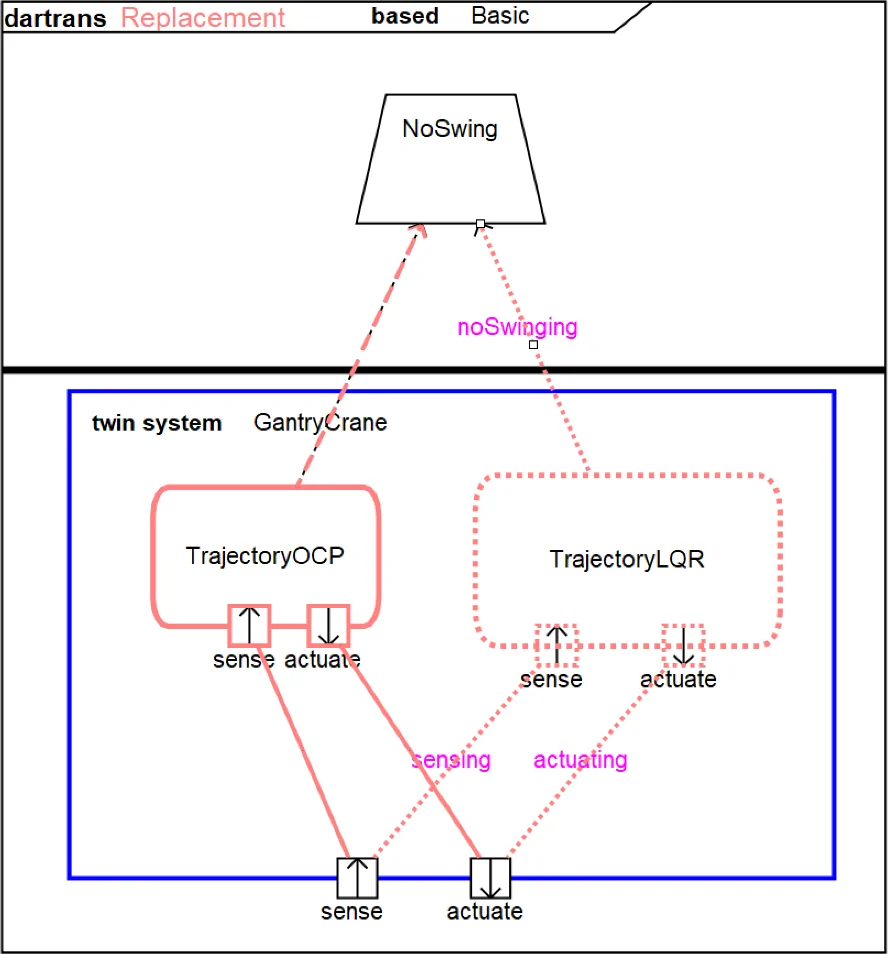
The result of merging the DarTwin into the DarTrans

In Fig. 21 we see that on the right side the elements to be replaced in the transition has been dotted. The Goal remains black. Furthermore, on the left side are the solid orange elements of the DarTrans and they have been supplied with additional information from our concrete environment. Here this amounts mostly to renaming the DT2 of the DarTrans to *TrajectoryOCP* which is the name of our new algorithm. Notice also that the name of the DarTrans is still *Replacement*. This is a small trick originating from our approach to setting the solid orange colour indicating new elements. All elements have a string that tells in what DarTrans they were included in the succession of evolutions. In Fig. 21 the solid orange elements have been copied from *Replacement* and therefore they are solid orange as long as the DarTrans is named likewise (Replacement). The next step is to change this.

**Fig. 22:**
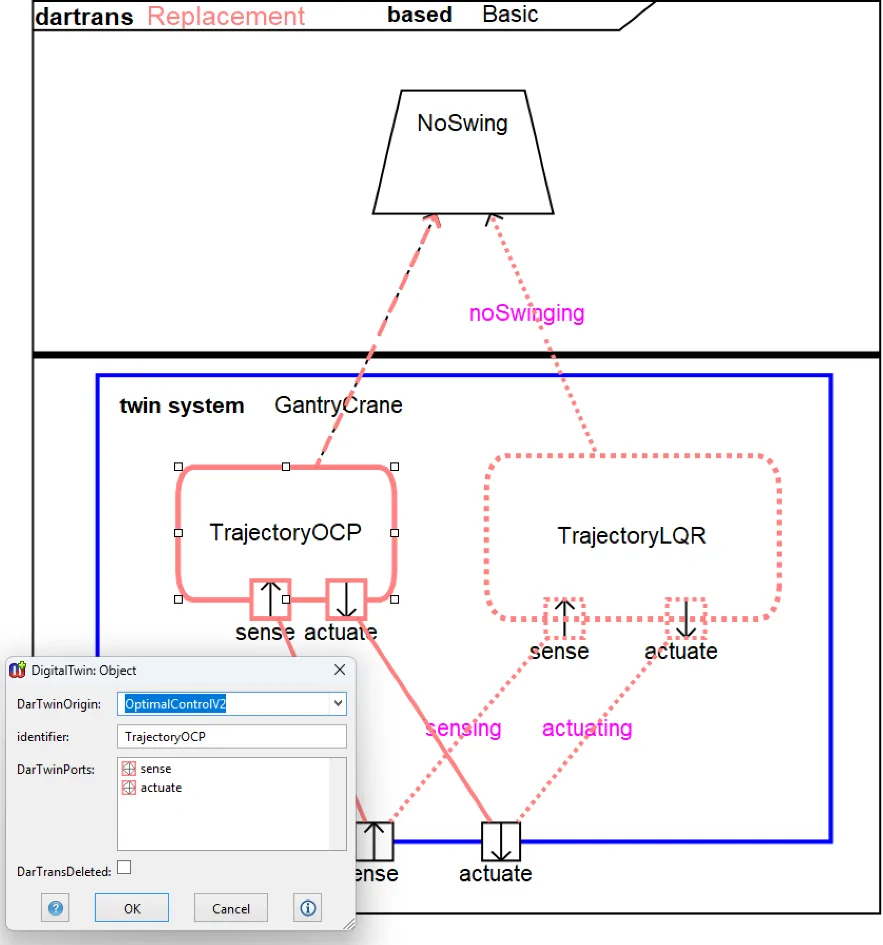
Alter the origin of the solid orange elements

We are now in the course of constructing a DarTrans that describes the concrete replacement evolution in the (simplified) Gantry system. That DarTrans will have the title *OptimalControlV2* and all its new elements should indicate with the dedicated string value that they were included in that. Therefore, we need to go through all the elements that are now solid orange and exchange “Replacement” by “OptimalControlV2” as the value of that dedicated origin property. For all elements that have been changed, the element will turn black. A snapshot of this process is shown in Fig. 22 (but with no elements having turned black, yet).

Now, we are almost there. The only thing we need to do now, is to rename the DarTrans itself to *OptimalControlV2* and that it is based on OptimalControl (first version).

**Fig. 23:**
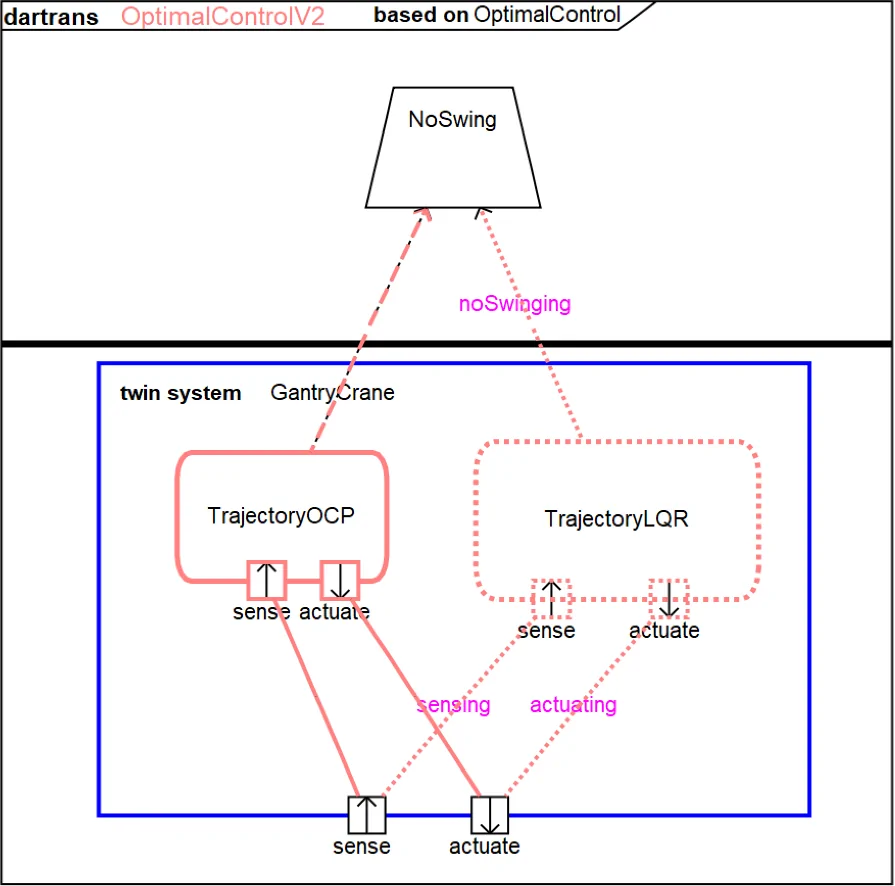
Rename the DarTrans to finalize the concrete evolution

The result can be seen in Fig. 23 which can be compared with Fig. 17. The two diagrams seem quite different at first glance, but looking closer it becomes clear that their DarTwin semantics are almost equivalent. The AT is in Fig. 17 represented by a part called PhysCrane while in Fig. 23 the AT is represented only by the ports on the twin system GantryCrane. The reader may recall that this difference is due to a bug in the Pilot Implementation. We also notice that the user-defined keywords are not done correctly in Fig. 17 while in Fig. 23 we do not need to use the DarTwin keywords because we have defined special DarTwin symbols. The colours should help to compare the diagrams since the solid orange, dashing and black correspond to the same elements.

**Fig. 24:**
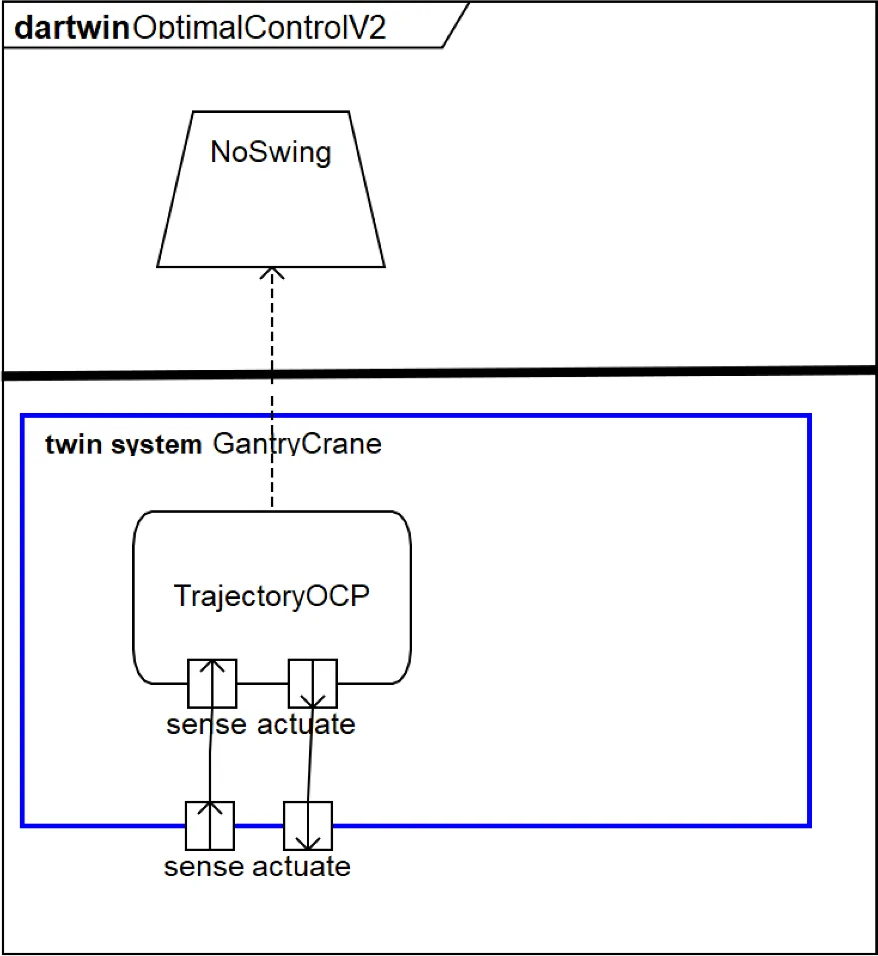
The concrete DarTwin for future evolutions

Now we have a description of our concrete evolution in the Gantry system, but our goal was to reach a more optimized Gantry, which means that we are only interested in our evolution for historical purposes. For the future, we want a clean DarTwin, and this can be achieved by removing all dotted orange elements and redefining the DarTrans to DarTwin as can be seen in Fig. 24 where all elements are now black.

Our question was whether MetaEdit+ tooling made the method more intuitive. Our own assessment is that the graphics with colours and dashes made the transition diagrams easier to overview and cope with than the textual listings. The necessary manual editing is also better supported by the way we have coded the colours in the MetaEdit+ tooling.

## DarTwin 3.0

The application of DarTrans transition patterns in the preceding section exposed limitations in the current DarTwin language. While the existing notation was sufficient for representing structural evolution transitions, it provided limited support for documenting why transitions occur, how they are initiated, and what broader evolution strategy they belong to. These observations motivated the exploration of additional DarTwin concepts related to triggers and transition strategies.

The competencies acquired when working with DarTwin and, in general, on DTs have motivated us to reconsider and enhance DarTwin once again. How should DarTwin 3.0 be improved, and how does this language improvement affect our current stack of worked examples, given that we apply MetaEdit+ and SysML v2?

The observation that usage experiences motivate improvements to the DSL is not new [23], and we also consider other needs that might arise shortly.

When working on the DarTrans method as described in Sect. 5 and exploring DTs and evolution in general as in [4], we realized that an evolution of DTs has its own characteristics beyond the description of the evolution itself with the before and after situations. Why did we initiate the evolution?

In [4], there is a table of *triggers* of changes to the DT systems. We think that it may be useful to be able to express this on a DarTrans diagram. Once we know why we want to evolve a DT system we have to find an evolution *strategy* and we think this could also be shown in the DarTrans.

Choosing the strategy for a concrete evolution is similar to choosing an appropriate DarTrans pattern if available. Associating strategy descriptions as well as trigger descriptions to the DarTrans patterns could make selecting the appropriate generic  easier. First the users would try and find a trigger that resembles the concrete trigger, and see quickly whether the starting point of the DarTrans seems fitting. Then the user would consider whether the pattern strategy could apply.

In the following, we augment the diagram on the applied DarTrans pattern Replacement from Fig. 19.

**Fig. 25:**
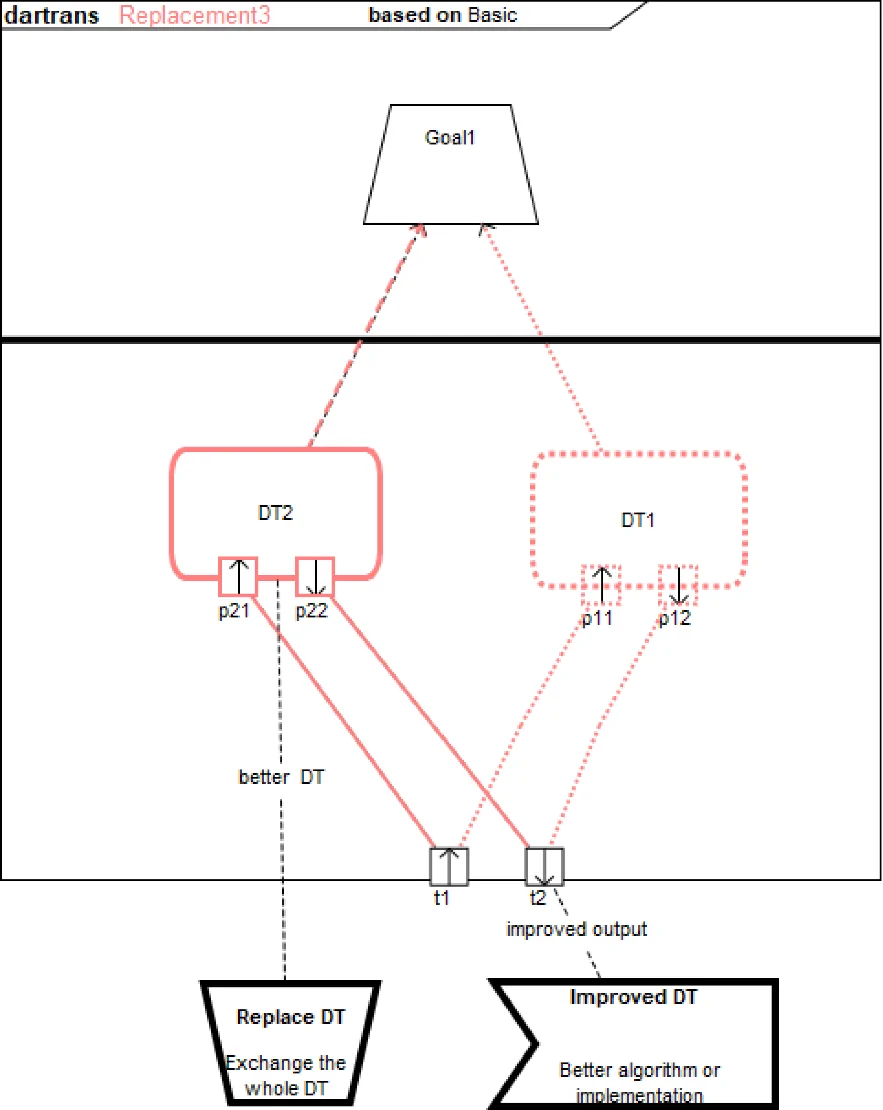
The *Replacement* DarTrans with new concepts intended to describe the trigger and the strategy for the transition

In Fig. 25 we have included a trigger (“Improved DT”) and a strategy description (“Replace DT”). We attach the trigger to the output port towards the AT to indicate that this pattern can be used for the purpose of improving the output, and the strategy attached to the DT2 shows that it recommends replacing the DT.

Our original Optimal Control in Fig. 18 was triggered by the promises of a new algorithm that would give even smaller amplitudes for the crane. This corresponds well with the trigger of *Replacement3* that refers to better algorithm for improvement. The strategy recommended from *Replacement3* is that the DT should be exchanged, and this seems a reasonable choice.

In our exploration of a DarTwin 3 version of our DSL we have also removed the twin system frame, as we find it redundant, making the diagram overly crowded. Implicitly, the twin system is now represented by the lower part of the DarTwin frame.

An advantage of using a DSL tool as the front-end is that experimenting with language changes can be done easily. We believe that additional concepts to DarTwin will need to be added to represent more of what concurrent research discovers about DT systems.

## Co-evolution analysis of the DarTwin toolchain

The preceding sections showed how evolving DarTwin required coordinated changes across graphical notation, SysML v2 definitions, generators, and existing models. These observations motivated analysing the DarTwin tooling ecosystem as a co-evolving multi-artifact environment. We frame these observations using the co-evolution taxonomy of Meyers & Vangheluwe [37] and the tool evaluation dimensions proposed by Tolvanen et al. [51].

### Domain and transition evolution in DarTwin

The transition from manually drawn DarTwin diagrams [36] to an embedded SysML v2-based DarTwin DSL  [24] constitutes *domain evolution*. The abstract syntax is formalized through a SysML v2 library and dedicated keywords, redefining conformance criteria and inducing secondary *model evolution* for existing artefacts.

The introduction of  further extends the language by explicitly modelling evolution steps via , , and . This is again domain evolution, but with stronger systemic consequences: models now encode structured transitions that must be interpreted consistently across tools.

In our multi-tool setting, the model-to-text generator from MetaEdit+ to SysML v2 constitutes a *transition artefact*. Consequently, every domain evolution induces *transition evolution*. Extending the language (e.g. adding triggers or refining goal concepts) requires coordinated updates to (i) the MetaEdit+ language definition, (ii) existing MetaEdit+ models, and (iii) the generator that serializes models into SysML v2. The generator embodies the assumptions of the current language version and is strictly one-directional; changes in generated SysML v2 artefacts cannot propagate back. This structural coupling makes generator maintenance a central co-evolution concern and introduces a risk of semantic drift if artefacts evolve out of sync.

### Tool support and co-evolution severity

Applying Tolvanen et al.’s evaluation dimensions clarifies where MetaEdit+ supports evolution effectively and where challenges remain. At the level of *concrete syntax*, MetaEdit+ enables relatively direct refinement of symbols and diagram styles. However, constraints related to uniform scaling and limited geometric control demonstrate that notation-level flexibility does not eliminate usability limitations in complex evolution narratives.

At the level of *abstract syntax* and constraints, changes exhibit higher severity. Issues such as repository-wide object scope and limited support for containment as a first-class concept affect multiple artefacts simultaneously: the language definition, existing models, and the generator. In this setting, co-evolution management becomes a cross-tool responsibility rather than a purely local modelling concern.

Overall, while MetaEdit+ provides strong support for notation evolution, it does not remove the need for systematic coordination across the wider toolchain. A central risk observed in our case is therefore not only tool instability, but divergence across artefacts when language, models, and generator evolve without explicit synchronization.

Taken together, these observations suggest that DarTwin evolution cannot be understood as isolated metamodel refinement. Instead, it unfolds as coordinated change across a coupled ecosystem of language definition, models, generators, and external tooling. Domain evolution systematically induces transition evolution, and the severity of change depends not only on syntactic modification but also on how artefacts are distributed across tool boundaries. This positions DarTwin as an illustrative case of multi-artefact co-evolution in practice.

### DarTwin language evolution

The challenge of model evolution in domain-specific languages has been recognized already early on in the design of such languages [44]. Many foundational and applied contributions have been made to the problem [50]. In essence, an evolution in a domain results in changes to the language definition, which in turn lead to models that might no longer conform to it. For our DarTwin, the evolution from DarTwin 1.0 to the future DarTwin 3.0 involves certain DT evolution concerns. The problem is further exacerbated when transformations are also part of the domain-specific ecosystem. In our case, DarTrans represents structured evolution transitions, while the presented model-to-text generation from MetaEdit+ to SysML v2 constitutes a separate transformation concern within the toolchain

To map the ramifications of evolution, Meyers and Vangheluwe present a framework for the evolution of languages, its models and transformations [37]. They classify the consequences of evolutions into four categories: (a) model evolution, when only the model changes but not its definitions, (b) image evolution, when the target language of a transformation evolves, (c) domain evolution, where the language definition changes, and finally (d) transformation evolution, where a transformation evolves independent of the language.

We see that the ongoing evolution of DarTwin is primarily located in category (c), since extensions such as triggers modify the language definition itself. However, these changes also introduce cascading co-evolution effects across the wider toolchain. In addition to evolving the MetaEdit+ language used for visualization, the model-to-text generator, responsible for producing SysML v2 artefacts, must also evolve to maintain consistency across representations.

When the language definition changes, all dependent artefacts must co-evolve to maintain conformance. The literature outlines three primary strategies for addressing this co-evolution challenge: manual specification of migration rules, metamodel matching techniques that infer migrations from differences between language versions, and operator-based approaches that record changes as reusable co-evolution operations [30, 31]. Most existing approaches focus primarily on restoring conformance between a modified metamodel and its instance models within a single tool environment.

In the context of DarTwin, however, the co-evolution problem extends beyond models alone to include graphical tooling, generators and external SysML v2 representations. In the future evolution of our DarTwin language, we will primarily use manual specification of migration rules.

Assessing the extent to which tools support the co-evolution of languages, models, and associated tools presents significant challenges. Tolvanen et al. [51] introduce an evaluation framework encompassing four dimensions: the location of change within the language definition (abstract syntax, constraints, or concrete syntax), the nature of the change (addition, renaming, removal, or modification), the affected components (other language definition parts, tools, generators, or existing models), and the severity of the impact. Applying this framework to MetaEdit+, Sirius, and Jjodel, they observe that all tools manage notation-level changes effectively, whereas metamodel changes present more substantial challenges for co-evolution across the entire artefact chain. The evaluation of MetaEdit+ is particularly pertinent to this study, as it offers an independent assessment of the tool used to implement DarTwin. Importantly, the framework treats generator co-evolution as a separate concern, which is consistent with the observation that evolving DarTwin necessitates updates not only to the MetaEdit+ language definition and models but also to the generator responsible for producing SysML v2.

## Related work

In this article we have introduced challenges related to language evolution, tooling interoperability, graphical notation support, and coordinated modification of models and generators. The related work reviewed here is not exhaustive, but it identifies adjacent areas that clarify what DarTwin does and does not aim to address. While these approaches are not specifically designed for representing evolution transitions in DT systems, they provide important conceptual and technical context for understanding how change can be modelled, communicated, and managed in complex engineering systems. Together, they help position DarTwin within the broader landscape of evolution-oriented modelling and systems engineering research.

In this paper, we have concerned ourselves with DarTwin —a language for describing evolution transitions in DT systems. Our focus has been on improving the precision of the language together with its graphical and textual tooling support through integration with SysML v2. Although evolution can generally be represented using behavioural or transition-oriented modelling approaches, our work is specifically targeted towards the representation and communication of evolution transitions in DT systems. Related fields nevertheless address comparable challenges of change and adaptation.

### Graph transformation, model transformation and DarTrans

Although the term *DarTrans* may suggest similarities to graph transformation approaches [14, 46], DarTrans is not intended as a graph transformation language in the formal model transformation sense. Traditional graph transformation approaches typically define executable rewriting rules operating on graph structures through formal matching and transformation semantics. In contrast, DarTrans models evolution transitions between DT system configurations using related before, core, and after representations intended to support communication, documentation, and systematic reasoning about DT system evolution. The purpose of DarTrans is therefore not automatic graph rewriting, but rather structured representation and application of evolution transitions in DT systems. This distinction is important for this article: DarTrans uses before/core/after structures to communicate evolution transitions, but it does not define transformation rules or an execution semantics for automatically rewriting models.

This positioning does not preclude future automation, and it is worth situating DarTrans relative to existing transformation work to clarify what such automation would entail. Czarnecki and Helsen [8] provide an overview of the features of model transformation languages, which would inform the design choices. For SysML v2, no dedicated graph-based transformation language is currently available.

In our case, the engineer manually selects the correct DarTwin as the starting point, and pattern matching and rewriting are performed manually. If automation of DarTrans application were pursued, the work that introduced T-Core [45] becomes relevant. T-Core presents a collection of transformation primitives, including pre- and post-condition patterns that allow specifying an evolution pattern, conceptually similar to how our DarTrans patterns describe before/after configurations. More of these transformation primitives would need to be considered.

Since SysML v2 is text-based, it also enables the use of text-based transformation languages, such as template-based approaches.

### Representing system and DT evolution

Several research areas address challenges related to system evolution, adaptation, and model change. These approaches address related challenges of system evolution and adaptation, and provide important conceptual context for DarTwin, which focuses specifically on representing evolution transitions in DTs.

The representation of system evolution has been addressed across several related research domains. While these fields often focus on runtime adaptation, software evolution, or system reconfiguration, they nevertheless address challenges that overlap with the goals of DarTwin Still, there are related fields of change that resemble our approach. Research on self-adaptive systems [10], for example, explores automated runtime reconfiguration in response to environmental and system changes, with MAPE-K loops [29] being among the most established approaches. In contrast, DarTrans does not focus on automated runtime adaptation, but rather on representing planned transitions between DT system configurations.

Beyond runtime adaptation, software and systems engineering research has long studied the broader problem of system evolution over time. Software system evolution has led to Lehman’s well-known *eight laws* [34], which have later been discussed in the context of software-intensive and Cyber-Physical Systems [53]. Many of these challenges are also relevant to DT systems, where systems continuously evolve through changing requirements, infrastructure, operational contexts, and integration needs. Such evolution may affect both the structure and purpose of DT systems, creating a need for approaches that can systematically represent, communicate, and reason about planned evolution transitions between different DT system configurations, which is the focus of DarTwin and DarTrans.

The DevOps of DTs can also be seen as natural evolution and has been studied in various ways in [7, 25]. These works primarily address architectural integration and interoperability rather than explicit representation of evolution transitions.

Some research addresses DT evolution more directly. [9] and [38] propose taxonomy-oriented frameworks for understanding DT evolution, while [3] presents a DevOps-oriented architecture framework intended to support DT evolution processes. However, these approaches either remain primarily conceptual or focus on operational infrastructure rather than explicitly representing concrete evolution transitions and their systematic application in DT systems, which is the primary concern of DarTwin and DarTrans.

### MDE for evolution, evolution for models

Variability and evolution has also been addressed in Model-Driven Engineering (MDE)-based domains in various forms. Dynamic Software Product Lines (DSPLs) [22] have been proposed to enable the binding/reconfiguration of variation points of SPLs at runtime. DarTwin instead focuses on representing planned evolution transitions in DT systems rather than runtime variability management.

SysML v2 natively supports the expression of *snapshots* [27], which may be used to describe a system’s state at a given time, comparable to the  and  (see e.g.  [20] and [5]). The concept, however, is limited to small-scale variations of property values, rather than large system reconfigurations. In comparison, DarTwin explicitly models broader evolution transitions between DT system configurations and their associated purposes.

Closely related to our work’s changes in purposes, we can look towards Architecture Description Languages (ADLs). [19], for instance, studied the automated evolution of AADL models based on requirements changes, and [49] studied the impact of changes in SysML v1 requirements. These works primarily focus on automated propagation and consistency management of model changes, whereas DarTwin focuses on representing and communicating planned evolution transitions.

Finally, we also studied the validity of systems and models throughout their lifetime using *validity frames* [12] and *modeling frames* [33]. These works introduce a framework for capturing the context in which modelling activities are performed, with the aim of improving reproducibility and re-usability. This notion of separating invariant and changing aspects of a system context conceptually aligns with the DarTwin distinction between stable and evolving parts of a DT system. The concept of a “frame” capturing what is invariant and what varies in a model context is conceptually aligned with the DarTwin distinction between , , and .

### DSLs, profiles, formalization

From a different viewpoint, DarTwin DSL relates to the development of DSLs, using SysML v2 as host language, using the natively provided means and mechanisms for customization. The concept of embedding DSLs in other host languages has been advocated for before [43]. UML, for instance, uses *profiles* [18] for adjusting the syntax and semantics. Unlike DarTwin in SysML v2, however, the semantics of UML profiles were not formally defined through UML itself. Notably, AADL further provides an *annex* mechanism that allows itself to be extended to add, e.g. discrete behaviour [16], continuous behaviour [2] or error modelling capabilities [11] to the language. These approaches primarily extend modelling expressiveness for specialized analysis concerns, whereas DarTwin focuses on representing evolution transitions and co-evolution concerns in DT systems.

This concept can also be extended to *internal* and *embedded* DSLs, which “use, and abuse” [17] programming languages as hosts, as shown by SystemC [6] and CREST [32], which use C++ and Python, respectively.

SysML v2 has already been targeted as the host language for domain-specification. In [15], the authors embed variability modelling capabilities within SysML v2. Like their variability-oriented DSL, our evolution-focused DarTwin DSL applies SysML v2 extensibility mechanisms to support precise modelling and systematic reuse. [26] and [1] explore the creation of domain-specific libraries.

Despite its extensibility, SysML v2 presents limitations that are directly relevant to our work. [27] analyses the language’s grammar and tooling, identifying gaps in modularity, semantics, and variant handling. These limitations motivate our decision to define a dedicated DSL within SysML v2 rather than relying solely on profiles or annotations for representing DT system evolution transitions.

## Conclusion & future work

This paper has told the story of how the DarTwin notation has evolved through usage, feedback and purposeful design. DarTwin was conceived as a graphical notation, and it has been developed into an embedded DSL with graphical tooling. We now have a workbench ready to support further developments of the language. Such development may be due to developments in the domain itself or in the technology supporting our DarTwin language. DarTwin is a language to describe the evolution of DT systems which currently constitutes an important research topic in DT engineering. Furthermore, our DarTwin tooling is partly based on that of SysML v2, which itself (and its tooling) undergoes evolution.

Our investigation revisits the three original hypotheses that motivated the DarTwin DSL from [24]. First, defining DarTwin as an embedded DSL in SysML v2 does indeed provide formal precision and well-defined semantics. Second, the DarTwin DSL is an extension of SysML v2 and as such the textual version of DarTwin integrates directly with SysML v2 and can be handled by SysML v2 tooling. Third, integration with SysML v2 tooling is technically feasible, but current SysML v2 tools do not yet preserve DarTwin ’s intended graphical abstraction. Our revisit to the tools did discover improvements, but even with manual post-editing the rendering did not correspond adequately with the DarTwin original notation. This motivated the exploration of a dedicated DSL workbench.

To mitigate the lack of adequate graphic support from SysML v2 tools, we presented the following findings for our exploration aims:

1. We investigated whether a dedicated DSL tool can better support the DarTwin evolution language while still preserving its formal alignment with SysML v2. SysML v2 tooling provides semantic precision and standards alignment, but does not provide DarTwin ’s intended graphical overview. Implementing DarTwin in MetaEdit+ restores full control over notation and diagram structure, but to preserve the connection with SysML v2, we program a generator in MetaEdit+ to produce textual SysML v2 that can be validated by SysML v2 tool such as the Pilot Implementation. We leave the MetaEdit+ definition from a textual DarTwin as future work.
2. Our formalization of DarTrans transitions further clarified the operational structure of evolution patterns. By encoding transitions explicitly through , , and , ambiguities present in earlier informal descriptions became visible and resolvable. The graphical realization in MetaEdit+ shows that evolution reasoning can benefit from visual traceability, while the SysML v2 encoding ensures semantic consistency.
3. Finally, the proposed DarTwin 3.0 extensions illustrate how practical usage feeds back into language evolution. The introduction of trigger and strategy concepts reflects the need to capture not only structural change but also the rationale and intent behind evolution decisions. This confirms that the DarTwin language, its tooling, and its transition methodology evolve together as an interconnected ecosystem.

Our experience, therefore, highlights that notation control, semantic precision, and tool integration must be treated as co-evolving concerns rather than independent design decisions.

Our findings are based on a single DSL and a limited set of tool configurations. Future work should assess the generality of the observed trade-offs across additional domain-specific extensions of SysML v2 and alternative DSL workbenches.

## Data Availability

No datasets were generated or analysed during the current study.

## Footnotes

1 However, in our examples that are also handled by the Pilot Implementation, we have added a part AT explicitly due to a rendering bug in the Pilot Implementation.

2 https://www.metacase.com/

3 https://plantuml.com/
